# Dementia with lewy bodies patients with high tau levels display unique proteome profiles

**DOI:** 10.1186/s13024-024-00782-0

**Published:** 2024-12-19

**Authors:** Sinead Greally, Mukesh Kumar, Christoph Schlaffner, Hanne van der Heijden, Elisabeth S. Lawton, Deeptarup Biswas, Sabina Berretta, Hanno Steen, Judith A. Steen

**Affiliations:** 1 F.M. Kirby Neurobiology Center, Department of Neurobiology, Boston Children’s Hospital, Harvard Medical School, Boston, MA 02115 USA; 2 Digital Engineering Faculty, Hasso Plattner Institute, University of Potsdam, Potsdam, 14482 Germany; 3 Harvard Brain Tissue Resource Center (HBTRC), McLean Hospital, Belmont, MA 02478 USA; 4 Department of Pathology, Boston Children’s Hospital, Harvard Medical School, Boston, MA 02115 USA

**Keywords:** Tau, α-synuclein, Neurodegeneration, Co-pathology, Proteomics, Mass spectrometry

## Abstract

**Background:**

Clinical studies have long observed that neurodegenerative disorders display a range of symptoms and pathological features and, in some cases, overlap, suggesting that these diseases exist on a spectrum. Dementia with Lewy Bodies (DLB), a synucleinopathy, is a prominent example, where symptomatic similarities with tauopathy, Alzheimer’s disease, are observed. Although tau pathology has been observed in DLB, the interplay between tau and α-synuclein is poorly understood at a molecular level.

**Methods:**

Quantitative mass spectrometry analysis was used to measure protein abundance in the insoluble fraction from cortical brain tissue from pathologically diagnosed DLB subjects (*n* = 30) and age-matched controls (*n* = 29). Using tau abundance, we stratified the DLB subjects into two subgroups termed DLBTau^+^ (higher abundance) and DLBTau^−^ (lower abundance). We conducted proteomic analysis to characterize and compare the cortical proteome of DLB subjects exhibiting elevated tau, as well as the molecular modifications of tau and α-synuclein to explore the dynamic between tau and α-synuclein pathology in these patients.

**Results:**

Proteomic analyses revealed distinct global protein dysregulations in DLBTau^+^ and DLBTau^−^ subjects when compared to controls. Notably, DLBTau^+^ patients exhibited increased levels of tau, along with ubiquitin, and APOE, indicative of cortical proteome alterations associated with elevated tau. Comparing DLBTau^+^ and DLBTau^−^ groups, we observed significant upregulation of cytokine signaling and metabolic pathways in DLBTau^−^ patients, while DLBTau^+^ subjects showed increases in protein ubiquitination processes and regulation of vesicle-mediated transport. Additionally, we examined the post-translational modification patterns of tau and α-synuclein. Our analysis revealed distinct phosphorylation and ubiquitination sites on α-synuclein between groups. Moreover, we observed increased modifications on tau specifically within the DLBTau^+^ subgroup.

**Conclusion:**

This molecular-level data supports the idea of neurodegenerative disease as a continuum of diseases with distinct PTM profiles DLBTau^+^ and DLBTau^−^ patients in comparison to AD. These findings further emphasize the importance of identifying specific and tailored therapeutic approaches targeting the involved proteopathies in the neurodegenerative disease spectrum.

**Supplementary Information:**

The online version contains supplementary material available at 10.1186/s13024-024-00782-0.

## Background

The idea of a continuum between pure tauopathies and synucleinopathies in neurodegeneration has gained support from clinical observations revealing high co-morbidity and overlapping clinical symptoms among patients [1, 2]. Dementia with Lewy Bodies (DLB) disease serves as a notable example of this continuum; it is classified as a synucleinopathy caused by the aggregation of the protein α-synuclein [3, 4]. However, patients diagnosed with DLB display significant symptomatic overlap with other neurological diseases like Alzheimer’s, the most prevalent tauopathy characterized by increased tau expression [5]. Evidence of tau pathology in the brains of patients with DLB has been reported through antibody-based analyses of post-mortem tissues, further corroborated by cerebrospinal fluid (CSF) analysis [6, 7]. However, tau and α-synuclein co-occurrence is not a universal feature of DLB patients, and only 20–50% demonstrate tau-positive pathology [8].

Nevertheless, the molecular changes underpinning the aggregated tau and α-synuclein in DLB are inadequately understood. It is even more critical to better understand the molecular nature of this disease continuum because of the emergence of tau- and α-synuclein potential therapeutics for tauopathies or synucleinopathies. Failure to identify patients with mixed pathologies could lead to the failure of clinical trials using these targeted approaches, as these individuals might respond significantly to treatments targeting a specific proteopathy. Therefore, utilizing quantitative mass spectrometry (MS) analysis, we explore the proteomics differences in a cohort of DLB patients, identifying individuals with significantly increased tau abundance in the cortex. Our investigation reveals the molecular distinctions of tau and α-synuclein and the potential disease pathways contributing to this phenomenon in patients with higher tau abundance.

## Methods

### Post-mortem human subjects and clinical data

For this data set, human post-mortem cortex specimens from DLB patients (*n* = 30), and age-matched controls (*n* = 29) were obtained from the Harvard Brain Tissue Resource Center (HBTRC) acquired through the NIH NeuroBioBank (U.S Department of Health and Human Services, National Institutes of Health). Control subjects had no clinical diagnosis of synucleinopathy, and DLB subjects fulfilled the pathological criteria for synucleinopathy disease. Neuropathological details for subjects are summarized in Table S1 and S2. Pathological and clinical information, if available, were de-identified.

### Sarkosyl fractionation of post-mortem tissue

A sarkosyl fractionation protocol was carried out to enrich the insoluble pathological α-synuclein and tau in all patients. Briefly, approx. 150–160 mg of post-mortem frozen cortical brain tissue per patient was homogenized in 640 µl TBS lysis buffer (50 mM Tris–HCL, 150 mM NaCl, 0.5 mM MgSO4, 10 mM ethylene diamine tetraacetic acid (EDTA), 10 mM ethylene glycol tetraacetic acid (EGTA), 1 mM Dithiothreitol (DTT), 10 mM Nicotinamide, 2 µM Trichostatin A, phosphatase inhibitor cocktail (Sigma) and protease inhibitor cocktail (Roche)), using Precellys® 24 tissue homogenizer (5500 speed, 3 cycles of 20 s with pause of 30 s). Homogenized tissue was clarified by centrifugation at 14,000 rpm for 20 min at 4 °C. The resulting supernatant (termed S1) was transferred to a new tube. The pellets were solubilized by adding 640 µl 1X Salt Sucrose Buffer (0.8 M NaCl, 10% sucrose, 10 mM Tris–HCL, 1 mM EDTA, 1 mM EGTA, 10 mM Nicotinamide, 2 µM Trichostatin A, phosphatase inhibitor cocktail (Sigma) and protease inhibitor cocktail (Roche)), and homogenized as previously, sonicated (1 min, 10 s on 5 s off, 20% amplitude) using a probe, and centrifuged. The resulting supernatant is termed S2, and the pellets are discarded. To S1, 640 µl of 2X Salt Sucrose Buffer was added, and too both S1 and S2 1% final concentration of sarkosyl was added. Samples were incubated for 1.5 h on the thermomixer (300 rpm at room temperature), and ultracentrifuged for 1.5 h (50,000 rpm at 4 °C). The resulting supernatants (sarkosyl soluble) were transferred to new tubes and the pellets (sarkosyl insoluble) were stored at −80 °C for further processing. Sarkosyl insoluble pellets are reconstituted in pellet buffer (50 mM Tris–HCL, 5% Sodium dodecyl sulfate (SDS), 10 mM Nicotinamide, 2 µM Trichostatin A, 8 M Urea, phosphatase inhibitor cocktail (Sigma) and protease inhibitor cocktail (Roche), 8 M Urea) and sonicated for analysis.

### Preparation of samples for mass spectrometry analysis

Following total protein concentration quantification with the bicinchoninic acid assay (Pierce™ BCA Protein Assay Kit, Thermo Scientific), 50 µg of total protein sample was digested using Single-pot, Solid-phase-enhanced sample preparation (SP3). Briefly, samples were diluted in 50 mM ammonium bicarbonate (ABC) and 8 M Urea buffer, 10 mM DTT was added to the samples and incubated for 30 min at 55 °C at 600 rpm. Alkylation was induced with the addition of 1% acrylamide and incubation at room temperature for 30 min. The alkylation reaction was quenched with the addition of 20 mM DTT and incubated at room temperature. Samples were loaded onto a combination of hydrophobic and hydrophilic magnetic beads (Cytiva LifeSciences) and binding was induced using 70% ethanol. Following incubation, the samples were washed with 2 washes of 80% ethanol, and 100% acetonitrile (ACN). Protein mixtures were digested with 12.5 ng/µl trypsin (sequencing grade modified trypsin, Promega, Madison, WI) for 16h at 37 °C. Digested peptides were removed from the magnetic beads and acidified using formic acid (FA). Peptides were desalted using C18 microspin columns (SEMSS18V, Nest Group, MA). Vacuum-dried peptides were reconstituted in MS sample buffer (5% FA, 5% ACN).

### LC–MS/MS measurements

Soluble and insoluble fractions of all samples were analyzed using the timsTOF Pro2 mass spectrometer coupled with ultra-high-pressure nano-flow liquid chromatography nanoElute system (Bruker, Germany). Peptides were loaded on to a reverse phase 25 cm aurora series C18 analytical column (25 cm x 75 µm ID, 1.6 µm C18) fitted with captive spray insert (Ionopticks, Australia). Column temperature was maintained at 50 °C and mobile phase A (2% acetonitrile, and 0.1% formic acid in water) and mobile phase B (0.1% formic acid in acetonitrile) was used for the separation of peptides with 400nL/min constant flow using a linear gradient starting from 0 to 30% in 90 min, followed by an increase to 80% B within 10 min, followed by washing and re-equilibration for 20 min. The mass spectrometer was operated in Data-Dependent Acquisition (DDA) mode using Parallel Accumulation Serial Fragmentation (PASEF). Full mass spectra were acquired within a mass range of 100–1700 m/z and an ion mobility (1/K0) range from 0.60–1.60. A top 10 PASEF method was employed, where 10 PASEF MS/MS scans were triggered per acquisition cycle for the most abundant precursor ions, followed by dynamic exclusion to avoid repeated fragmentation of the same precursor.

### Immunohistochemistry and analysis

Chromogenic immunohistochemistry was used to detect phosphorylated tau and alpha synuclein. Five-micron thick sections were cut from formalin-fixed paraffin-embedded sections from the same Dorsolateral Prefrontal cortex Brodmann area 9 (BA9) from 16 donors from the DLB cohort. The sections were mounted on slides to produce four slides per donor. Slides were deparaffinized and rehydrated with xylenes and graded alcohol. After antigen unmasking with a citrate buffer and incubating in Peroxidized 1 (Biocare Medical, cat#PX968), slides were blocked with Background Punisher (Biocare Medical, cat#BP974). Two sections per donor were incubated in anti-human PHF-Tau AT8 (Thermo Scientific, 1:400, mouse monoclonal, cat#MN1020), and two sections per donor were incubated in purified anti-a-Synuclein LB509 (Sigma Millipore, 1:4000, mouse monoclonal, cat#MABN824). Slides were then incubated in Mach 4 Mouse Probe HRP Secondary antibody, followed by incubation in Mach 4 HRP polymer (Biocare Medical, micro-polymer detection, cat# M4U534L). All slides were then visualized with DAB peroxidase substrate, counterstained with hematoxylin, cleared, and mounted.

Brightfield whole slide scans were performed using an Olympus VS120 Slide Scanner at 20 × magnification (Neurobiology Imaging Facility, Boston, MA). A semi-automated quantitative analysis of phosphorylated tau expression was performed on each image using HALO™ Image Analysis software (Indica Labs, Albuquerque, NM). Quantification of tau positive cells across all images of tau-stained slides was carried out using the HALO™ IHC Multiplex module. The IHC Multiplex module was tailored with customized parameters, a custom-trained nucleus segmentation classifier, and a custom-trained quality control tissue classifier. A total cell count was acquired by counting all nuclei positive for hematoxylin. Quantification of tau positive cells was shown as the number of tau positive cells over the total cell count (hematoxylin positive). Additionally, the images were analyzed using the HALO™ Area Quantification Module tailored with the same custom-trained nucleus segmentation classifier and custom-trained quality control tissue classifier. Quantification of tau expression was shown as the percentage tau positive tissue area over the total section area. Quantification of α-synuclein positive cells across all images of α-synuclein stained slides was also performed using the HALO™ Area Quantification Module tailored with a custom-trained nucleus segmentation classifier and custom-trained quality control tissue classifier. Quantification of α-synuclein expression was shown as the percentage α-synuclein positive tissue area over the total section area.

### Multiplex immunofluorescence and Imaging

Formalin-fixed paraffin-embedded (FFPE) cortical brain tissue sections from one DLBTau⁻ and one DLBTau⁺ patient were used for immunofluorescence analysis. Five-micron thick sections were cut and mounted on slides. Tissue sections were deparaffinized using xylene and rehydrated through graded ethanol into distilled water. Antigen retrieval was performed by heating the slides in citrate buffer (pH 6.0) at 95 °C for 20 min, followed by cooling to room temperature. To prevent non-specific binding, slides were blocked with 5% normal goat serum in PBS for 1 h at room temperature.

The sections were incubated with the following primary antibodies, each diluted at 1:100: recombinant Alexa Fluor® 488 anti-α-synuclein aggregate (Abcam, cat# ab216124, clone MJFR-14–6-4–2), Alexa Fluor® 647 beta-amyloid (Cell Signaling Technology, cat# D3D2N), and Alexa Fluor® 750 phospho-tau (T217) (Cell Signaling Technology, cat# E9Y4S). DAPI (Cell Signaling Technology) was used as a nuclear stain. All sections were incubated with the primary antibodies overnight at 4 °C in a humidified chamber.

Following incubation, slides were washed with PBS and mounted with an anti-fade mounting medium. Fluorescence imaging was performed using the Advanced Solutions BioAssemblyBot 200 CELL DIVE Automation workstation and the CELL DIVE multiplex imaging system (Leica Microsystems). Images were acquired at 20 × magnification using the CELL DIVE Mx-workflow software, which applied automatic image corrections including autofluorescence removal, distortion correction, blank glass subtraction, and flat-field correction. Fluorescent signals were detected at AF-488 nm for α-synuclein, AF-647 nm for beta-amyloid, and AF-750 nm for phospho-tau.

Image analysis and visualization were conducted using AIVIA software (version 14.1, Leica Microsystems). Brightness and contrast were adjusted, and images were captured at full slide size, as well as at 100 μm and 50 μm resolutions, for figure preparation. Co-localization analysis of the different fluorescent markers was performed to evaluate the spatial distribution of α-synuclein aggregates and phospho-tau.

### Software, statistical analysis and data visualization for proteomic data

DDA timsTOF data files were analyzed using Fragpipe (version 17.1) with MSFragger (version 3.4) for proteome analyses, and MASCOT software (version 2.6.1) for PTM identification. Briefly, raw data was analyzed in MSfragger, and peptide list searched against the Homo sapiens Uniprot protein sequence database (January 2021). The following parameters were applied: a minimum of two unique peptides per protein for identification, stricttrypsin cut at lysine and arginine with up to two missed cleavages, mass tolerance set to 20 ppm. Oxidation of M, acetylation of N-terminal and K, phosphorylation of S,T,Y, and ubiquitination of K were chosen as variable modifications and propionylation of cysteine as fixed modification. False discovery rate (FDR) was set to 1% on peptide and protein levels with a minimum length of six amino acids. For all other search parameters, the default settings were used.

All statistical analyses were performed using Perseus (version 2.0.5.0), GraphPad Prism (version 10.1.12), Excel (version 2408) and Cytoscape (version 3.9.1) with the STRING and STRING Enrichment applications. For differential expression analysis in Perseus, protein groups were filtered to remove contaminants, reverse hits, and those identified only by site. Using the MSFragger output files, label-free protein quantification intensity values were Log2-transformed to normalize the wide range of protein abundance values and facilitate statistical analysis. Proteins were filtered for a minimum of 70% valid values in at least one treatment group, and missing values were not imputed. Differentially expressed proteins were identified using a t-test with a permutation-based false discovery rate (FDR) threshold of < 0.05. Volcano plots for figures were generated in GraphPad Prism.

Box-and-whisker plots, created in GraphPad Prism, were used to display the Log2 intensities of proteins of interest, showing maximum and minimum values per group, with statistical significance between cohorts assessed by Student’s t-test (*p***-**value < 0.05). Log2-transformed tau protein intensities were used for clustering analysis, where a classical k-means clustering with two clusters was performed using Orange (version 3.36.2), initialized with the k-means + + method and followed by 50 re-runs and 1000 iterations. A 2D scatter plot with jittering was generated to reduce overplotting, based on cluster number and Log2 intensities. Excel was used to create histograms for peptide intensities in proteins of interest.

Pathway enrichment analysis was conducted using Cytoscape with the STRING Enrichment plugin, focusing on Gene Ontology (GO) Biological Pathways. Final figures were prepared using BioRender (app.biorender.com).

### Western blot analysis

Pooled protein samples were prepared from all groups: controls (CT, *n* = 30), DLBTau⁻ (*n* = 21), and DLBTau⁺ (*n* = 9). For each group, 10 µg of pooled protein was mixed with Invitrogen™ NuPAGE™ LDS Sample Buffer (4X) and Invitrogen™ NuPAGE™ Sample Reducing Agent (10X). Samples were denatured at 70 °C for 10 min using a thermomixer. Each sample (20 µL) and Invitrogen™ SeeBlue™ Plus2 Pre-stained Protein Standard were loaded onto 10-well 4–20% Mini-PROTEAN TGX Precast gels (Bio-Rad). The gels were run for approximately 1 h at a constant voltage of 150 V.

Proteins were transferred onto PVDF membranes using the Bio-Rad Trans-Blot Turbo Transfer System for 7 min. The membranes were blocked and subsequently incubated overnight at 4 °C with the following primary antibodies: APP/β-Amyloid (NAB228, 1:500, mouse monoclonal, cat#2450, Cell Signalling Technology), Clusterin (D4B6P, 1:500, rabbit monoclonal, cat#42,143, Cell Signalling Technology), EGF Receptor (D1D4J XP®, 1:500, rabbit monoclonal, cat#54,359, Cell Signalling Technology), and Ubiquitin (P4D1, 1:500, mouse monoclonal, cat#3936, Cell Signalling Technology). Membranes were washed and incubated with Invitrogen™ IRDye 680 goat anti-rabbit and IRDye 800 donkey anti-mouse secondary antibodies.

Total protein was assessed using the LI-COR Revert™ 700 Total Protein Stain, and imaging was performed on a Bio-Rad ChemiDoc MP Imaging System. Total protein was visualized at IRDye 680, while the primary antibodies were detected at IRDye 680 (for rabbit) and IRDye 800 (for mouse).

Images were analyzed using ImageJ software (version 1.53c). The area under the total protein lanes and specific bands of interest were calculated. The intensity of the bands of interest was normalized to the total protein stain for each lane. Ratios of protein intensities were calculated by comparing the normalized intensities to that of the control group. For presentation, the images were adjusted for brightness and contrast and cropped for Supplementary Fig. 5. Bar graphs were created using GraphPad Prism (version 10.1.12) and depict normalized intensities and ratio intensities for all antibodies of interest, representing one replicate per group, with each group being a pooled sample.

## Results

### The workflow to study tau and α-synuclein in human DLB patients using MS-based proteomics

**Fig. 1:**
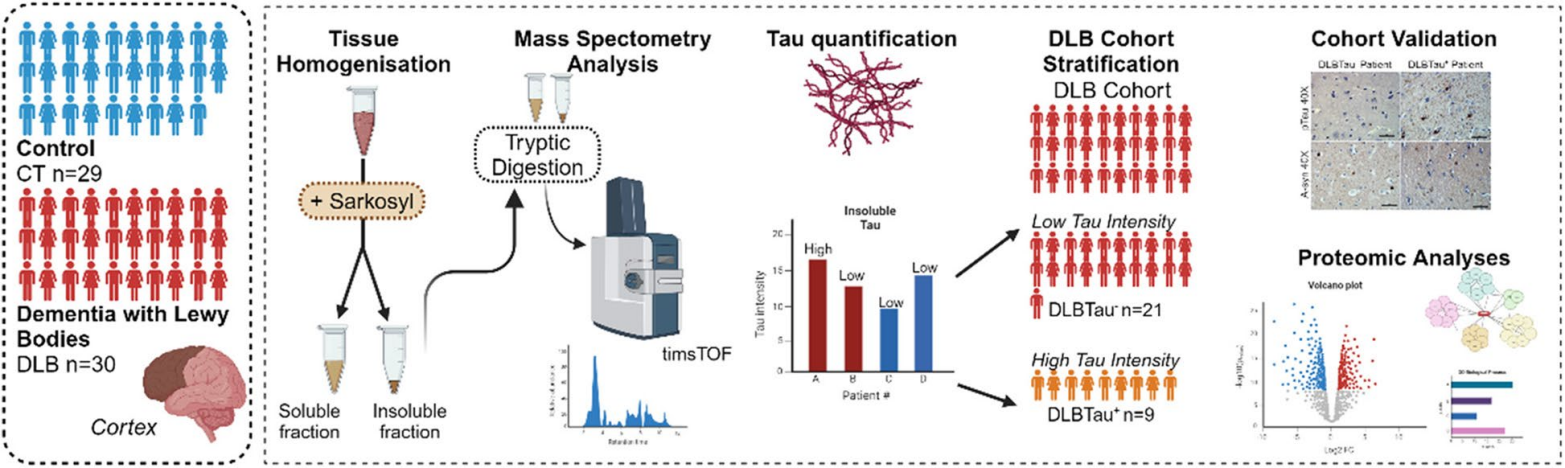
Study workflow and stratification of DLB cohort based on insoluble tau levels in cortical brain tissue. Cortical brain tissue from cohorts of aged healthy control (CT) (*n* = 29) and DLB patients (*n* = 30) were subjected to sarkosyl fractionation and subsequent tryptic digestion. Soluble and insoluble fractions were analyzed on the timsTOF Pro2 mass spectrometer using a 90-min gradient. The MS measurement of tau abundance was used to stratify the DLB cohort based on higher or lower abundance. Histological staining validated cohort subgrouping, and further proteomics analyses in the DLBTau^−^ (*n* = 21) and DLBTau^+^ (*n* = 9) groups were performed

We provide the workflow and rationale of the study in Fig. 1. Fresh-frozen cortical brain tissue was used from a cohort of age-matched human subjects, including healthy control subjects (CT, *n* = 29) and Dementia with Lewy Body patients (DLB, *n* = 30), who were diagnosed using post-mortem pathology. The tissues were acquired from the McLean/Harvard Biobank, and associated metadata for these human subjects are provided in Supplementary Table S1 and S2. The tissues were subjected to protein extraction using an optimized MS-compatible sample preparation involving sarkosyl fractionation to enrich the pathological and aggregate form of tau and α-synuclein. The resulting soluble and insoluble sarkosyl fractions were digested using trypsin and analyzed on a timsTOF Pro2 mass spectrometer using data-dependent acquisition methods. Label-free protein quantification identified a significant increase in insoluble tau protein in a subset of the DLB cohort. This led to a stratification of DLB subjects based on tau abundance and the two groups were subsequently labeled as DLBTau^−^ (low tau abundance) and DLBTau^+^ (high tau abundance). This increased tau aggregation was validated using immunohistochemistry and immunofluorescence, and further analyses were performed to determine if the insoluble tau and α-synuclein were distinct in the DLBTau^+^ patients from the DLBTau^−^ patients. Global proteomics data was also collected and examined to determine if differentially regulated proteins/pathways are associated with the increased tau abundance with α-synuclein pathology.

### Stratification of the DLB patient cohort using MS-measured tau abundance

**Fig. 2:**
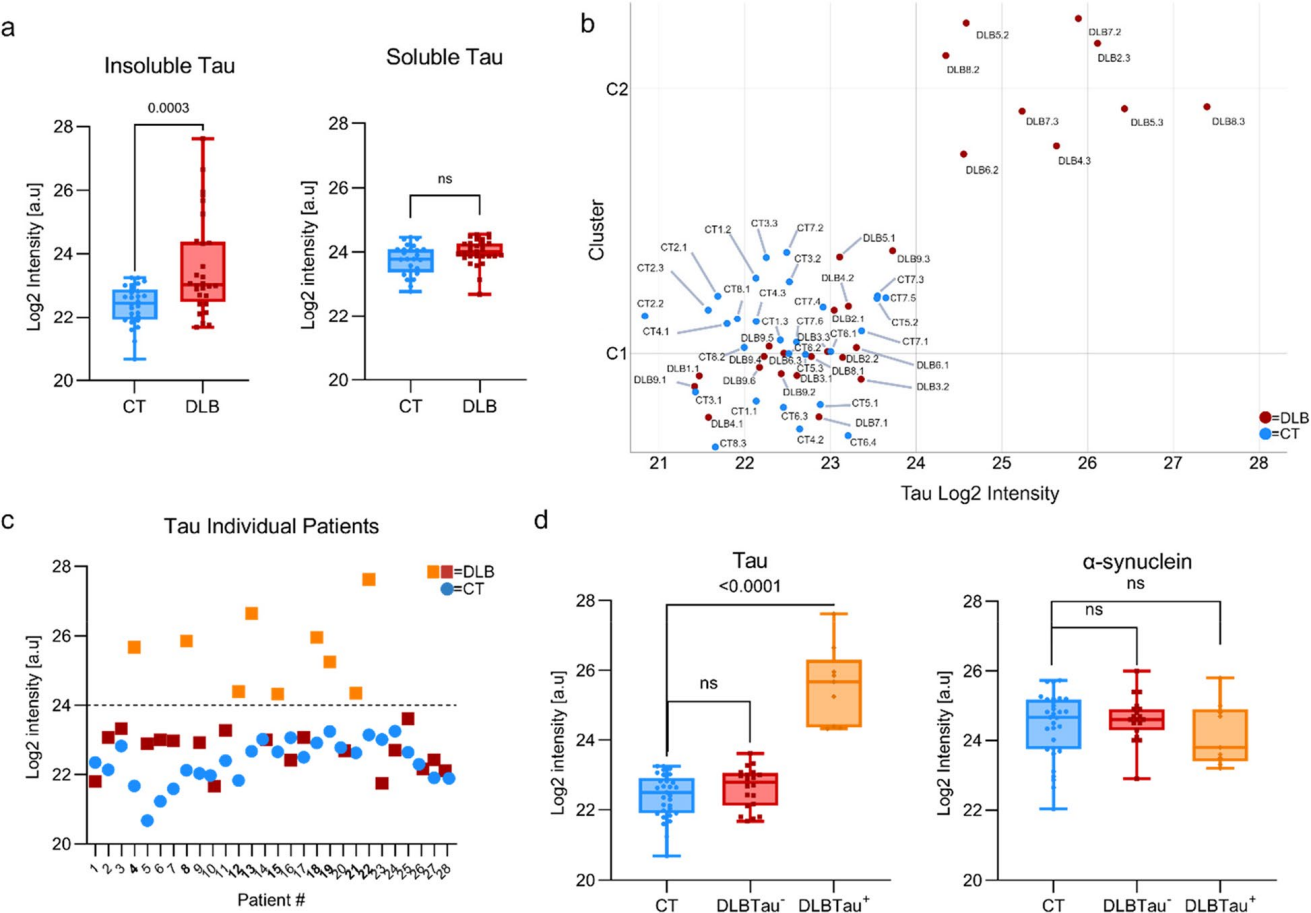
MS analyses stratify DLB patients based on tau abundance. **a** Tau abundance is significantly upregulated in the insoluble fraction but not the soluble fraction of cortical brain tissue in a DLB cohort (*n* = 30) versus controls (CT) (*n* = 29). **b** K**-**means clustering of the DLB patients revealed two distinct groups based on tau abundance, with a cutoff at a Log2 intensity of 24. **c** Individual tau intensities of all patients in the DLB and CT cohorts indicate 9 DLB patients with higher-than-control tau levels. **d** DLB cohort’s subgrouping based on the tau intensities confirmed increased insoluble tau in the DLBTau^+^ patients (orange) versus DLBTau^−^ patients (red) and controls (blue). Insoluble α-synuclein levels are comparable between all groups

We first identified a significant increase of insoluble tau abundance in the DLB group compared to CT, which led to the question of whether all DLB subjects displayed this increased expression. While insoluble tau is significantly higher, soluble tau does not differ significantly in the DLB group relative to controls, aligning with tau abundance commonly observed in tauopathies [9] (Fig. 2a). To assess the individual tau intensities per patient, we used the individual Log2 intensities per patient and carried out a clustering analysis. A k-means clustering analysis was performed with two clusters (CT and DLB) using Log2-transformed tau intensities to generate a 2D scatter plot. This analysis identified 9 DLB patients with tau intensities clearly separating at a Log2 value greater than 24 (Fig. 2b). This was further replicated by plotting the individual tau abundance Log2 intensities in Fig. 2c, with DLB patients with higher tau shown in orange, while the remaining 21 patients shown in red showed levels consistent with controls. The 9 DLB patients had an average tau abundance (Log2 intensity) of 25.5 and a median of 25.6, while the remaining 21 DLB subjects showed lower tau levels with an average of 22.6 and a median of 22.7. These levels were similar to the control group, which had an average and median tau abundance of 22.4. Based on these findings, the DLB cohort was stratified into two subgroups: DLBTau^+^ (higher tau intensities, *n* = 9) and DLBTau^−^ (lower tau intensities, *n* = 21). When separated, tau (insoluble) intensity was significantly higher in the DLBTau^+^ group relative to controls and the DLBTau^−^ group. However, notably, insoluble α-synuclein intensities remained comparable across all groups (Fig. 2d).

### Tau and α-synuclein show areas of co-localization in a DLBTau^+^ patient

**Fig. 3:**
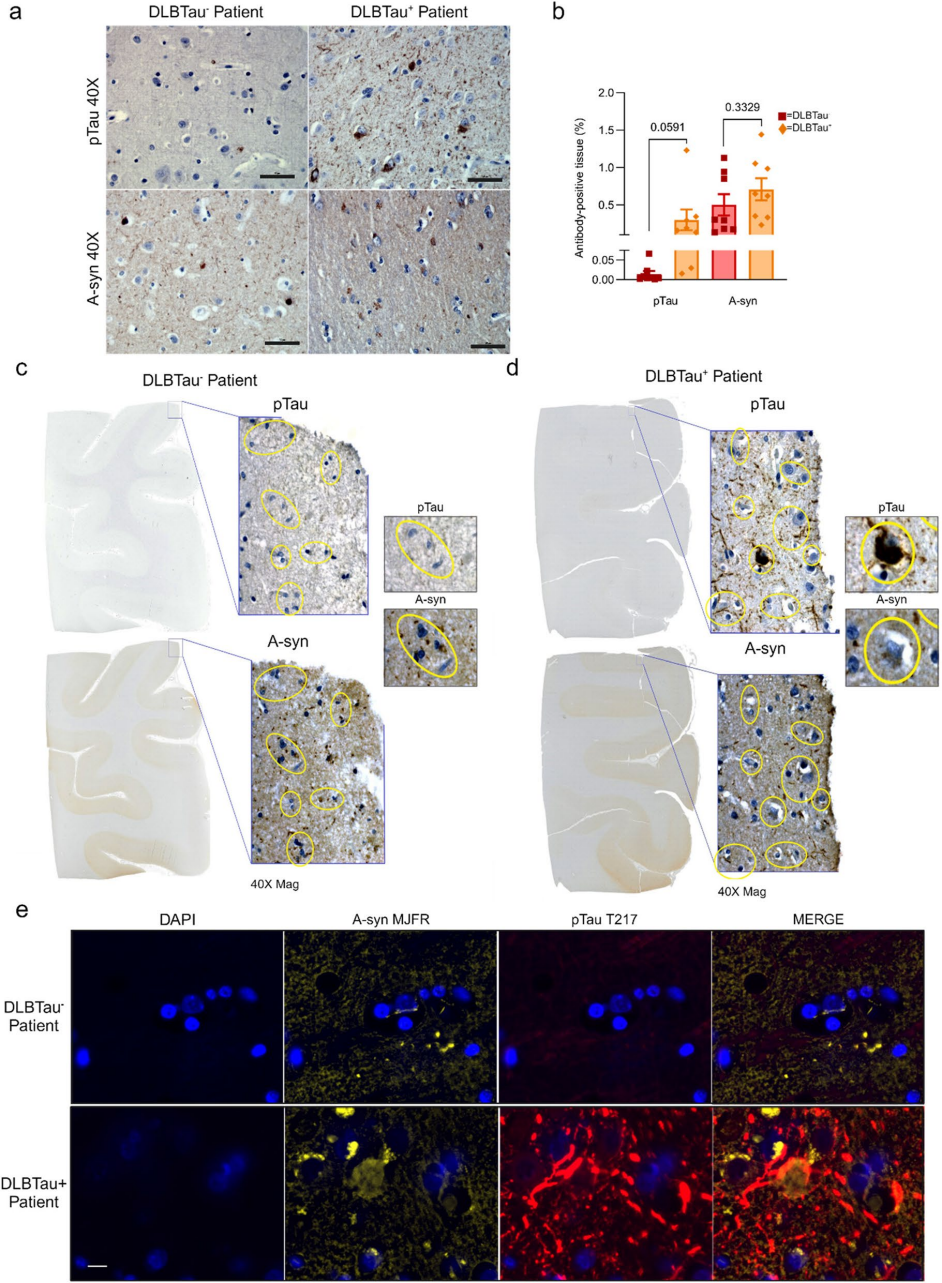
IHC and IF analyses of DLBTau^+^ patients confirm increased tau abundance and show co-localization with α-synuclein. The nuclei are stained blue (hematoxylin), and the pTau and a-syn antibodies are brown (DAB peroxidase substrate) (**a**) Immunohistological staining of cortical brain tissue of one DLBTau^+^ and one DLBTau^−^ patient as defined previously, showing phosphorylated tau (pTau) positive staining in the DLBTau^+^ patient as well as total α-synuclein staining, indicating co-pathology of aggregates of both proteins. The DLBTau^−^ patient showed no pTau staining. **b** Quantification of percentage of tau-positive tissue, and α-synuclein-positive tissue, in the DLBTau^+^ patients versus DLBTau^−^ patients. **c-d** Adjacent 5 µm cortical slides were visualized using hematoxylin nuclei, pTau, and α-synuclein staining in one DLBTau^−^ (**c**) and one DLBTau^+^ (**d**) patients with highlighted overlapping cells (yellow) show co-localization of aggregates of both tau and α-synuclein in the DLBTau^+^ patient. **e** Multiplex IF of cortical tissue showing α-synuclein aggregates (yellow), phosphorylated tau (red), and DAPI-stained nuclei (blue). The DLBTau⁻ patient exhibits no detectable pTau, while α-synuclein aggregates are present. In the DLBTau⁺ patient, pTau and α-synuclein co-localize near DAPI-positive nuclei, indicating intracellular aggregation of both proteins. Images have been captured at × 40 magnification for IHC and × 20 magnification for IF; scale bars represent 50 μm (black) and 10 μm (white)

An orthogonal method to measure tau, i.e., immunohistochemical (IHC) analysis using a phosphorylated tau (pTau AT8) antibody, confirmed the existence of tau aggregates in our DLBTau^+^ cohort, an example of one patient classified as DLBTau^+^ is shown in Fig. 3a, while a DLBTau^−^ patients exhibited no signs of tau pathology. The increase in tau expression was additionally confirmed through quantification with the percentage of tau-positive tissue per patient. Additionally, IHC analyses of both patient groups displayed aggregates of α-synuclein using the anti- α -synuclein LB509 antibody, and quantification revealed no significant differences between the groups (Fig. 3b) (individual quantification for pTau and α -synuclein shown in Supplementary Fig. 1). To assess co-localization of tau and α-synuclein pathology, we performed both IHC staining and multiplex immunofluorescence (IF) staining on cortical tissue from one patient in each group. Adjacent 5 µm sections from both a DLBTau⁻ and DLBTau⁺ patient were stained using the same tau and α-synuclein antibodies as previously employed for IHC quantification. The pTau and α-synuclein antibodies showed aggregates of both proteins in overlapping areas of the tissue in the DLBTau^+^ patient, with no evidence of tau co-staining present in the DLBTau^-^ patient (Fig. 3c and d). For multiplex imaging, using the CELL DIVE system, α-synuclein aggregate-specific antibody MJFR and phosphorylated tau pTau T217 were used. The images revealed an absence of pTau staining in the DLBTau⁻ patient, while α-synuclein aggregates were clearly present in the cortical tissue. In contrast, the DLBTau⁺ patient exhibited extensive pTau staining, which co-localized with α-synuclein aggregates near DAPI-positive nuclei, suggesting aggregation within cellular bodies (Fig. 3e).These analyses confirm the co-occurrence of tau and α-synuclein aggregates in the DLBTau^+^ group and validated our DLB subgrouping based on proteomic quantification.

### DLBTau^+^ and DLBTau^−^ patients exhibit distinct cortical proteome differences

**Fig. 4:**
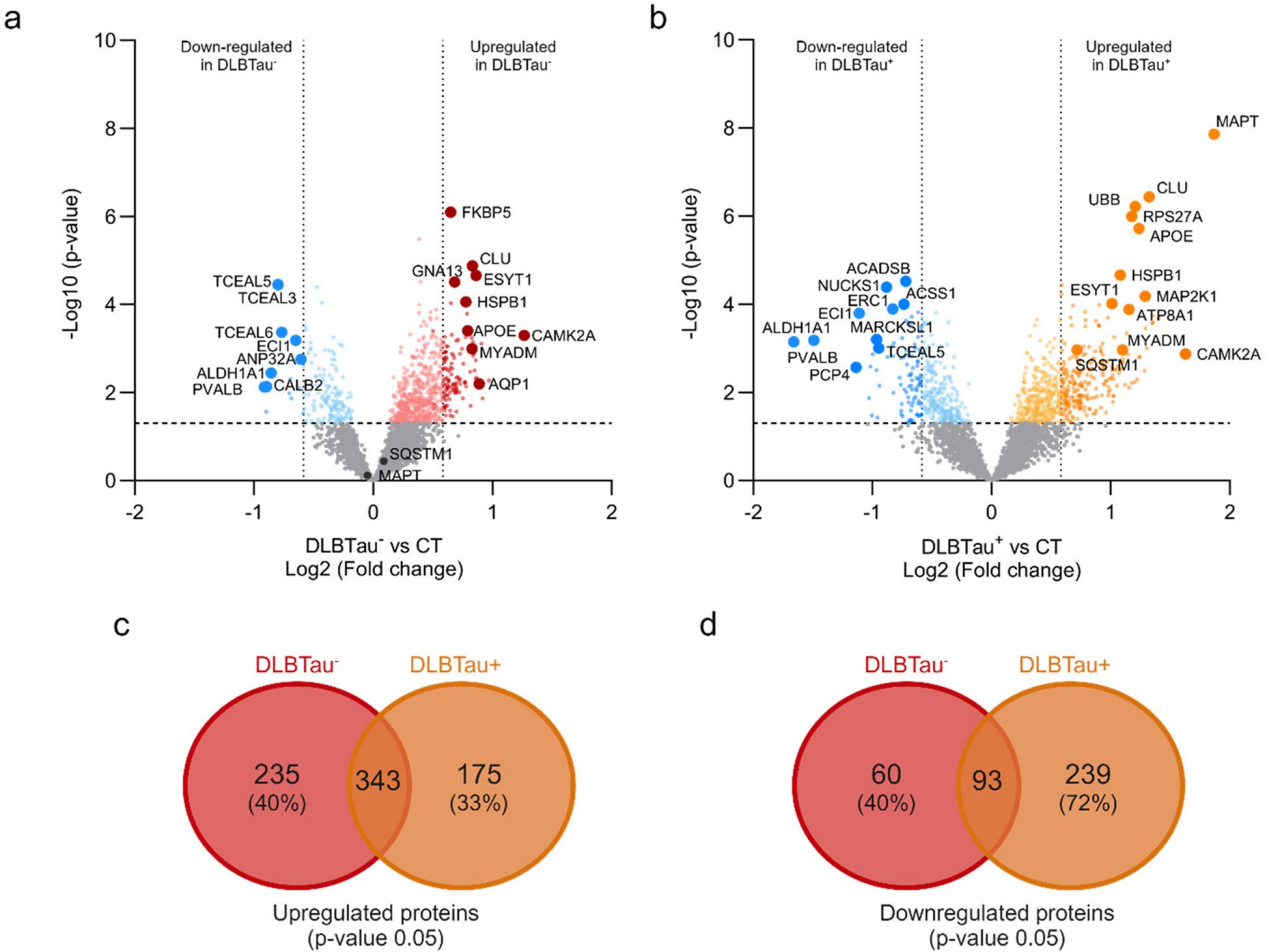
Proteome changes between DLB subgroups and aged controls. **a** The volcano plot depicts the global proteome analysis of the cortical insoluble fraction, revealing an upregulation of 630 total proteins in the DLBTau^-^ patients versus controls and a downregulation of 156 total proteins. Among the 630 upregulated proteins, 145 also passed the multiple correction with an FDR of < 0.05, (dark red), and out of 156 downregulated proteins, 23 passed multiple correction with an FDR of < 0.05, (dark blue). Lines show an unadjusted -Log10 *p***-**value of 1.3 (*p***-**value < 0.05) and Log2 fold change of < −0.585 and > 0.585 for fold change (FC) cut-off > 1.5. **b** The volcano plot depicts the global proteome analysis of the cortical insoluble fraction, showing an upregulation of 581 total proteins in DLBTau^+^ patients and a downregulation of 346 total proteins. Among the 581 upregulated proteins, 124 also passed the multiple correction with an FDR of < 0.05, (dark orange), and out of 346 downregulated proteins, 57 passed multiple correction with an FDR of < 0.05, (dark blue). Lines show an unadjusted -Log10 *p***-**value of 1.3 (*p***-**value < 0.05) and Log2 fold change of < −0.585 and > 0.585 for fold change (FC) cut-off > 1.5. **c-d** Venn Diagram of the number of overlapping and unique upregulated and down-regulated proteins in DLBTau^+^ and DLBTau^−^ subjects compared to controls

Independent comparisons of DLBTau^−^ subjects (*n* = 21) compared to controls (*n* = 29) (Fig. 4a) and DLBTau^+^ (*n* = 9) compared with control subjects (Fig. 4b) show significant proteomic alterations in the insoluble fraction of the cortical tissues. Some of these dysregulated proteins were common to both DLBTau^−^ and DLBTau^+^, including Calcium/calmodulin-dependent protein kinase type II subunit alpha (CAMK2A), apolipoprotein E (APOE), apolipoprotein L2 (APOL2), extended synaptotagmin 1 (ESYT1), and myeloid associated differentiation marker (MYADM), all of which showed increased abundance in both subgroups compared to controls (FDR-adjusted *p***-**value < 0.05). For both DLBTau^−^ and DLBTau^+^ patients, clusterin (CLU), a major genetic risk factor for late-onset Alzheimer’s disease (AD), also exhibited a higher expression (FDR-adjusted *p***-**value < 0.05) and was the second most elevated protein in both subgroups [10]. However, the most significantly altered protein differed between the subgroups. In DLBTau^-^ subjects, FKBP5 (FKBP Prolyl Isomerase 5) was the most highly upregulated protein compared to controls (FDR-adjusted *p***-**value < 0.05). This was striking, as this protein has not been identified in DLB previously but has been implicated in stress physiology and may contribute to inflammation with increased aging [11, 12]. In contrast, in DLBTau^+^, tau (MAPT) was the most significantly upregulated protein compared to controls (FDR-adjusted *p***-**value < 0.05). Interestingly, several proteins involved in the ubiquitin–proteasome system (UPS) were also elevated in DLBTau^+^, including sequestosome protein (SQSTM1), and ubiquitin protein (UBB), none of which showed significant changes in DLBTau^−^ patients.

In both subgroups, we identified common proteins with reduced expression, such as transcription elongation factors (TCEAL3/5/6), aldehyde dehydrogenase 1 (ALDH1A1), and parvalbumin (PVALB), all of which were significantly downregulated compared to controls (FDR-adjusted *p***-**value < 0.05). However, distinct downregulation patterns were observed within each group. In DLBTau^+^, several acyl-CoA dehydrogenases/synthetases (ACADSB, ACSS1) and kinases, such as NUCKS1 (nuclear casein kinase) and MARCKSL1 (myristoylated alanine-rich C-kinase substrate), were also significantly reduced in abundance (FDR-adjusted *p***-**value < 0.05). In contrast, transcription elongation factors were the most significantly decreased in DLBTau^−^ compared to controls (FDR-adjusted *p***-**value < 0.05) (Fig. 4a and b). Volcano plots depicting the proteome analysis of the corresponding soluble cortical fractions of DLB subgroups versus controls showed similar trends (Supplementary Fig. 2).

Using a Venn diagram, we present the number of differentially expressed proteins that are either uniquely dysregulated or shared between the DLB subgroups. Our findings reveal that among the upregulated proteins in DLBTau^-^ subjects, 40% are unique, with 343 shared proteins, whereas in DLBTau^+^ subjects, 33% of the upregulated proteins are unique (Fig. 4c). For downregulated proteins, 40% of the downregulated proteins in DLBTau^-^ patients are unique as observed with upregulated proteins. In contrast, a significant proportion of the downregulated proteins in DLBTau^+^ patients (72%) are unique (Fig. 4d). This analysis reveals that, even though both groups were diagnosed with DLB disease, the presence of increased tau expression correlates with a marked change on the cortical insoluble proteome in these patients.

### Significantly dysregulated proteins and pathways in DLBTau^+^ subjects

**Fig. 5:**
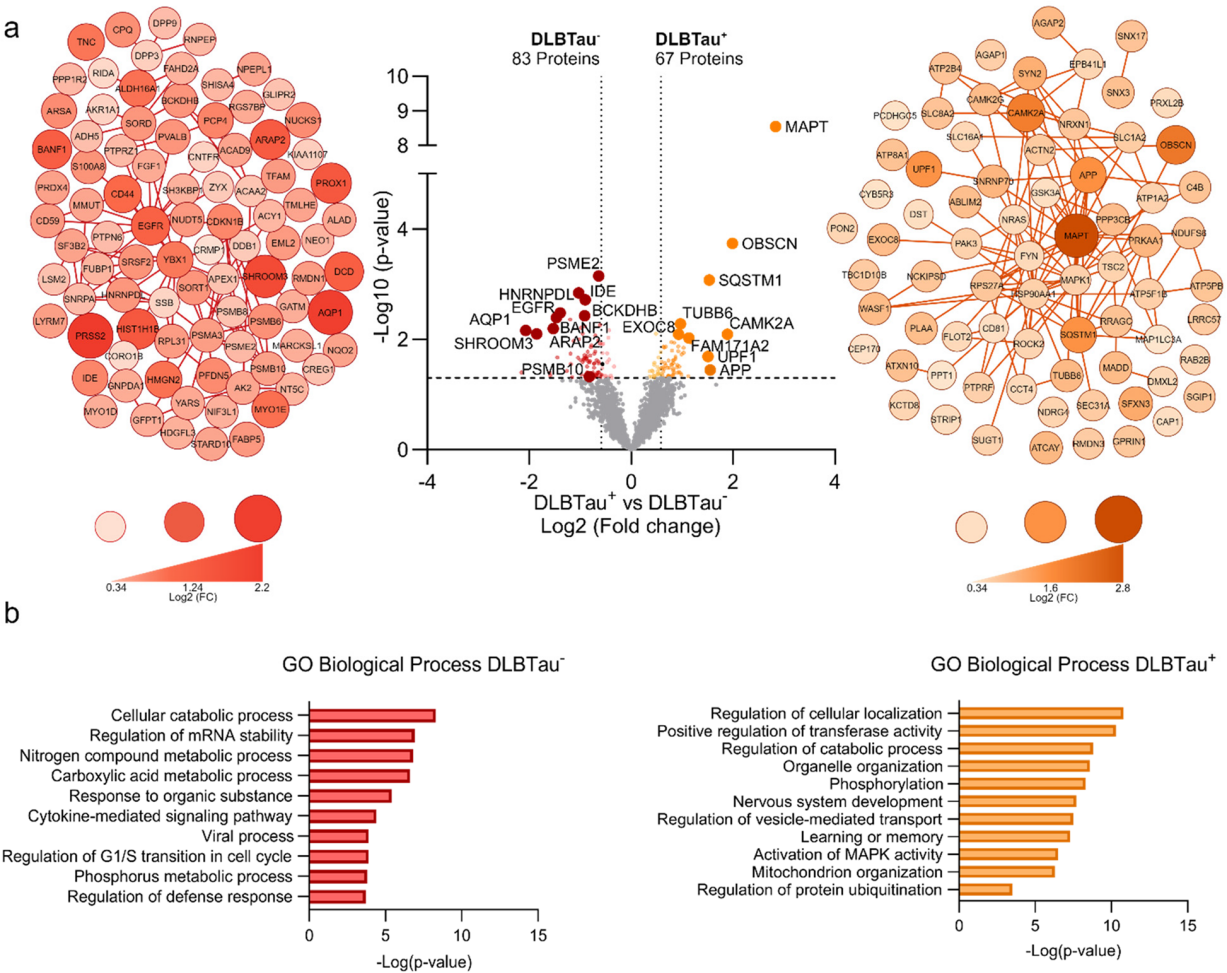
DLBTau^+^ patients show differential protein expression and pathways compared to DLBTau^−^. **a** Volcano plot depicting differential proteome analysis between the cortical insoluble fraction of DLBTau^+^ and DLBTau^−^ patients showing the upregulation of 67 proteins in DLBTau^+^ patients, including MAPT, SQSTM1, and APP, and an upregulation of 83 proteins in the DLBTau^−^ patients including AQP1, EGFR and immunoproteasome-associated proteins. The dotted lines show an unadjusted **-**Log10 *p***-**value of 1.3 (*p***-**value < 0.05) and Log2 fold change of < −0.585 and > 0.585 for fold change (FC) cut-off > 1.5. The network node color intensity and size are indicative of a Log2 fold change (Log2 FC) between 0.34 to 2.2. A network analysis of the total upregulated proteins in DLBTau^+^ patients is shown, node color and size range from a Log2 fold change (Log2 FC) between 0.35 to 2.8. **b** GO Biological process enrichment analysis revealed 17 GO Biological pathways are associated with the 83 proteins upregulated in DLBTau^−^ patients, and the top 10 significant pathways are shown here. Over 100 pathways are associated with the upregulated proteins in DLBTau^+^ patients, showing the top 10 significant pathways and the protein ubiquitination pathway

Following this, we compared the distinguishing proteins and pathways between each DLB subgroup. Among nearly 3,000 identified proteins, 67 were significantly elevated in the DLBTau^+^ patients (based on unadjusted *p***-**value), with tau being the most significantly increased in this group. Other proteins include dementia-associated proteins such as amyloid precursor protein (APP) and SQSTM1 protein, a protein associated with ubiquitination (Fig. 5a).

We aimed to investigate whether, in addition to the overall APP protein being upregulated, amyloid**-**β**-**specific peptides within the amyloid-β region were also elevated. From our MS data, we identified and used three sets of amyloid-β-specific peptides, both tryptic and semi-tryptic, encompassing the Aβ_1–42_ isoform, which is considered the major component in Alzheimer’s disease plaques [13]. Using these peptides, we found that amyloid-β was significantly upregulated in both DLB subgroups, with the specific Aβ_1–42_ peptides showing additional upregulation in DLBTau⁺ patients compared to DLBTau⁻. This upregulation of amyloid-β in patients with higher tau levels was further corroborated by IF staining of cortical tissue from a DLBTau⁻ and a DLBTau⁺ patient, where stronger amyloid-β staining was observed in the DLBTau⁺ sample. (Supplementary Fig. 3).

The DLBTau^−^ group showed 83 proteins with increased abundance (based on unadjusted *p***-**values), including immunoproteasome-associated proteins PSME2, PSMB8, and PSMB10, as well as the tyrosine-kinase receptor EGFR, which has been implicated in the prion-like propagation of α-synuclein, and aquaporin (AQP1) [14]. While some proteins showed significant differences between DLBTau^+^ and DLBTau^−^ based on unadjusted *p***-**values, none were significant after FDR correction (FDR < 0.05), which is expected since both are subgroups of the same cohort. Volcano plots displaying the proteome analysis of the soluble cortical fractions from DLBTau⁺ and DLBTau⁻ groups showed comparable results (Supplementary Fig. 4).

We conducted a network analysis visualizing the significantly upregulated proteins associated with DLBTau^−^ and DLBTau^+^ subgroups, utilizing size and color gradients to convey LogFC (fold-change) (Fig. 5a). GO Biological Process enrichment analysis was carried out on the significantly changed proteins in both groups. Biological processes enriched with the 83 DLBTau^−^ proteins include catabolic, metabolic, and cytokine-mediated signaling pathways. Over 100 significant pathways were associated with the 67 upregulated proteins in DLBTau^+^ patients, including regulation of cellular localization, nervous system development, mitochondrion organization, and protein ubiquitination (Fig. 5b). The distinct proteins and pathways identified in DLBTau^+^ patients may play a role in the increased levels of tau or could result from the aggregation of both tau and α-synuclein.

We validated key proteomic findings, observing consistent up**-** and downregulation patterns across DLB subgroups for APP, UBB, EGFR, CLU, and PSMB10 through Western blot analysis with accompanying images and quantification. To further confirm these findings, we analyzed peptide-level intensities for APOE, CLU, EGFR, PSMB10, SQSTM1, and UBB using a semi-tryptic MSFragger search, averaging intensities per peptide and visualizing them in Supplementary Fig. 5. Histograms with 2**-**period moving average trendlines for CT, DLBTau^-^, and DLBTau^+^ groups highlight similar trends to those in the full proteomic dataset, protein quantification, and pooled Western blot data (Supplementary Fig. 5).

### Tau and α-synuclein PTM profiles are distinct in DLBTau^+^ and DLBTau^−^ patients

**Fig. 6:**
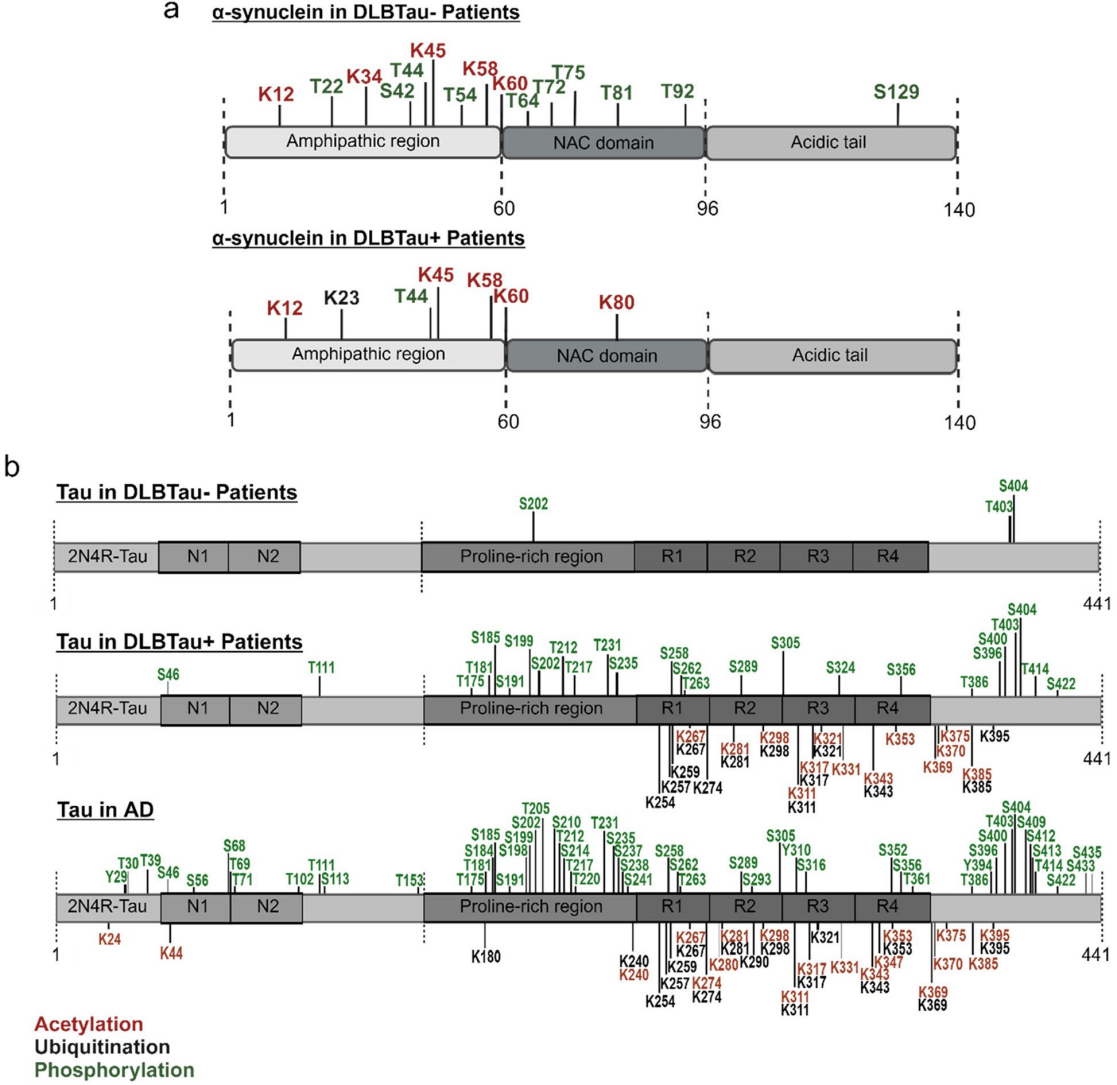
PTM profiles of α-synuclein and tau between DLB subgroups. **a** Cumulative map of the PTMs identified on α-synuclein in DLBTau^+^ and DLBTau^−^ patients showing numerous phosphorylation sites in patients without increased tau abundance. Patients in the DLBTau^+^ group showed a site of ubiquitination at K23 on the α-synuclein. **b** Tau PTMs landscapes identified in DLBTau^+^ and DLBTau^−^ patients and comparing them to a typical cumulative map of PTMs in AD. Tau identified in patients in the DLBTau^−^ group displayed minimal phosphorylation and no other modifications, while DLBTau^+^ patients had extensive phosphorylation, ubiquitination, and acetylation extending from the proline-rich region to the end of the protein. Tau in a cumulative map in AD displayed extensive modifications from the N- to C-terminal of the protein in comparison

Post-translational modifications (PTMs) of both tau and α-synuclein play crucial roles in synucleinopathies and tauopathies, potentially influencing their aggregation and contributing to the neurodegenerative pathogenesis [15, 16]. However, there is a significant gap in studies identifying PTMs of these proteins in patients with tau and α-synuclein aggregation, particularly utilizing MS analysis. A comparison of cumulative PTMs identified on α-synuclein reveals notable differences between the DLBTau^+^ and DLBTau^−^ groups. α-synuclein in the DLBTau^−^ group exhibited more phosphorylation sites, including phosphorylation of serine 129 (pS129), a recognized hallmark of synucleinopathies [17]. Both groups showed multiple acetylation sites on α-synuclein, primarily at the N-terminal, with DLBTau^+^ α-synuclein featuring an additional site at K80 (acK80) within the mid-domain. Furthermore, α-synuclein in DLBTau^+^ patients displayed fewer phosphorylation sites but had increased ubiquitination at K23 (ubiK23) (Fig. 6a). Upon analysis of tau modifications, we found stark differences between the subgroups. The DLBTau^−^ patients tau exhibited minimal modifications of phosphorylation at three sites (pS202, pT403, and pS404), while tau in DLBTau^+^ patients displayed 26 phosphorylated sites identified mainly in the proline-rich region. Along with phosphorylation, the increased level of ubiquitination and acetylation of tau was also observed in this group. This starkly contrasted to the tau in DLBTau^−^ patients that displayed no acetylation and ubiquitination. The patient frequencies of identified PTMs of both α-synuclein and tau in each DLB subgroup are provided in Supplementary Table S3.

To determine if the tau in DLBTau^+^ patients is similar to that of a tauopathy, we compared the tau PTM landscape to a cohort of AD patients previously analyzed by our lab [9]. This comparison reveals that tau in DLBTau^+^ patients is less extensively modified than in AD. Differences include the absence of phosphorylation and acetylation at the N-terminal in DLBTau^+^ patients, reduced phosphorylation sites at the C-terminal, and the identification of one site of phosphorylation at S324, not found on AD tau in that study (Fig. 6b). Additionally, we wanted to determine if the PTM profile in DLBTau^+^ patients mirrored that of early-stage AD. In Supplementary Fig. 6, we separated the early, middle, and late stages of AD and found that the DLBTau^+^ patients did not display the same PTM landscape as early-stage AD. Instead, DLBTau^+^ tau exhibited a unique profile that did not mirror any of the stages in AD.

## Discussion

The concept of a disease spectrum encompassing synucleinopathies and tauopathies has gained significant attention recently [18]. Using a cohort of DLB subjects, we stratified patients based on mass-spectrometry measurement of insoluble tau abundance, irrespective of pathology findings or Braak staging, which traditionally rely on specific tau antibodies targeting certain modifications and may not fully quantify total tau expression [19]. This is of particular note as the tau in DLBTau^+^ subjects exhibit a different PTM profile to AD tau. Our comprehensive proteomic analysis revealed notable proteome differences in the cortex between patients identified in this study as DLBTau^+^ and DLBTau^−^, including dysregulated proteins and pathways that distinguish the subgroups. Interestingly, compared to controls, we observed ubiquitin-associated proteins like sequestosome or p62 protein and the ubiquitin protein as some of the top significantly upregulated proteins in the DLBTau^+^ patients. When compared to DLBTau^−^ patients, the protein ubiquitination pathway was upregulated significantly in these patients, along with more dementia-related proteins like amyloid-β, specifically the pathogenic Aβ_1–42_ isoform. The exact involvement of the ubiquitination system in synucleinopathies or tauopathies is not fully understood but has been significantly implicated in both protective and toxic contributions to disease pathology [20–23]. Whether ubiquitination has a role in the co-occurring pathology of tau and α-synuclein adds an extra complexity.

This increased ubiquitination was also identified in the PTM profiles of both proteins in the DLBTau^+^ patients, with the tau displaying several sites of this modification, as well as acetylation and phosphorylation. However, comparison to tau in typical tauopathies, including various AD stages previously characterized by our lab, shows that tau in DLBTau^+^ patients presents a unique PTM profile, with fewer N-terminal modifications, unlike those seen in early AD [9]. These unique differences emphasize that DLB patients with increased tau abundance may represent a novel disease entity on the synucleinopathy and tauopathy disease spectrum, as they do not resemble typical DLB synucleinopathy or AD tauopathy.

This understanding holds significant importance, particularly because of the increasing use of biologics particularly antibodies as therapeutics for these diseases targeting specific proteoforms of α-synuclein and tau [24, 25]. Our study emphasizes the additional stratification for patients of synucleinopathy or tauopathy diseases co-occurence, who could be excluded or placed in a different group in clinical trial exclusion for trials targeting only tau or synuclein. This could potentially improve clinical trial outcomes as a patient with co-pathologies may not respond to all tau antibody therapies nor will the therapy address the synucleinopathy pathology. Further, this stratification could identify patients with co-pathology, presenting an opportunity for unique therapeutic strategies. This study also highlights the potential use of current or new tau/α-synuclein body fluid biomarkers.

The coexistence of tau and α-synuclein in patients, like our DLBTau^+^ group, still invokes several questions: whether these co-pathologies are independently initiated, how they affect each other or how they may contribute or exacerbate disease progression and, therefore, associated symptoms. Utilizing proteomics to stratify patients with neurodegenerative disease and identifying mixed pathologies of both tau and α-synuclein could potentially identify underlying mechanisms contributing to the phenomenon. By using proteomic analysis to quantify the tau abundance in the brain, we were able to stratify our patients using an unbiased approach. The results highlight the opportunities to add CSF or plasma evaluations of tau and α-synuclein to categorize patients for a more accurate diagnosis and therefore a specialized therapeutic approach and better prognosis. This could then enable the stratification of living patients by identifying targets to develop positron emission tomography (PET) reagents or associated biomarkers in body fluids in future studies.

## Conclusion

In conclusion, this study highlights the potential use of unbiased proteomic approaches to stratify synucleinopathy patients and characterize the proteome distinctions in patients with increased tau abundance. The distinct molecular pathways and PTM profiles of tau and α-synuclein in DLBTau^+^ patients suggest a unique disease entity on the spectrum of synucleinopathy and tauopathy. Our study underscores the importance of personalized therapeutic strategies tailored to individual molecular profiles.

## Supplementary Information

Supplementary Material 1.

Supplementary Material 2.

Supplementary Material 3.

## Data Availability

Additional data is summarized in Additional Files including Supplementary Excel File 1 summarizing all proteomic results including protein intensities and statistical results. Mass-spectrometry data will be made available on PRIDE upon acceptance of this manuscript for publication.

## Abbreviations

DLB: Dementia with Lewy Bodies
AD: Alzheimer’s disease
CT: Controls
PTM: Post-translational modification
CSF: Cerebrospinal fluid
MS: Mass spectrometry
SP3: Single-pot, solid-phase-enhanced sample preparation
DDA: Data-dependent acquisition
IHC: Immunohistochemistry
pTau: Phosphorylated tau
FDR: False discovery rate
CAMK2A: Calcium/calmodulin-dependent protein kinase type II subunit alpha
APP: Amyloid-precursor protein
APOE: Apolipoprotein E
APOL2: Apolipoprotein L2
ESYT1: Extended synaptotagmin 1
MYADM: Myeloid associated differentiation marker
CLU: Clusterin
FKBP5: FKBP Prolyl Isomerase 5
MAPT: Tau
UPS: Ubiquitin-proteasome system
SQSTM1: Sequestosome protein
UBB: Ubiquitin protein
TCEAL3/5/6: Transcription elongation factors
ALDH1A1: Aldehyde dehydrogenase 1
PVALB: Parvalbumin
ACADSB: Acyl-CoA dehydrogenases
ACSS1: Acyl-CoA synthetases
NUCKS1: Nuclear casein kinase
MARCKSL1: Myristoylated alanine-rich C-kinase
APP: Amyloid precursor protein
PSMB8/9/10: Immunoproteasome subunit8/9/10
EGFR: Epidermal growth factor receptor
AQP1: Aquaporin
