# Supplementary material for: Dementia with lewy bodies patients with high tau levels display unique proteome profiles
Source: Mol Neurodegener. 2024 Dec 19;19:98. doi: 10.1186/s13024-024-00782-0 (PMC11657859; doi:10.1186/s13024-024-00782-0)
Supplement: Supplementary file 1 — Supplementary Material 1. [file 13024_2024_782_MOESM1_ESM.zip › Supplementary Figure 1.docx]

Supplementary Figure 1


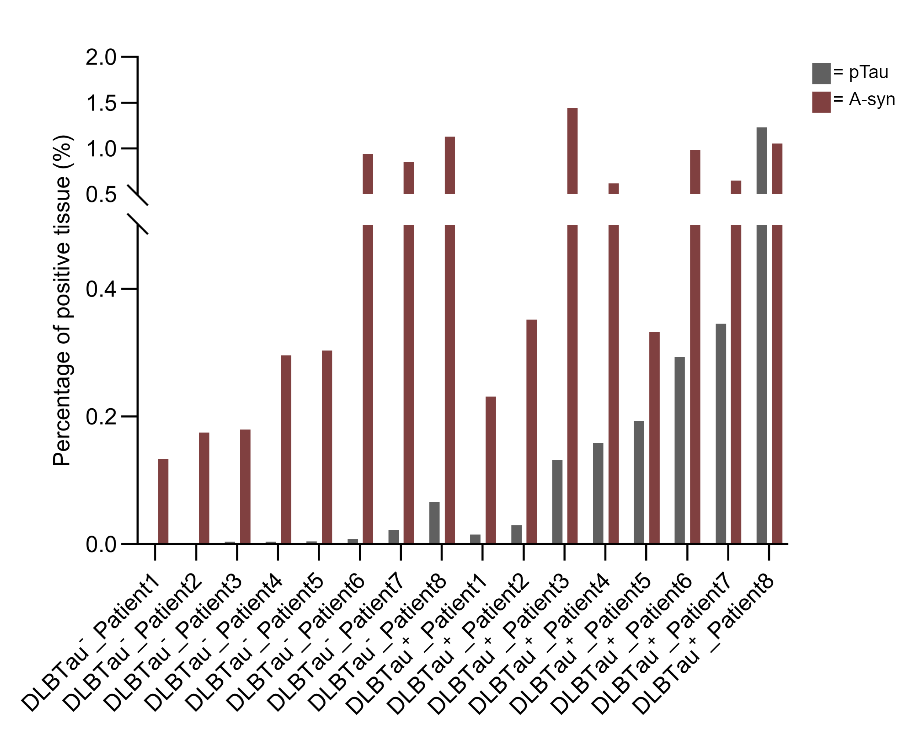


**Supplementary Figure 1. Percentage of cortical tissue positive for pTau and α-synuclein antibodies for 8 individual DLBTau^-^ and DLBTau^+^ patients.** Bar chart depicting the quantified percentage of tissue positive for both pTau, phosphorylated tau (grey bars), and A-syn, total α-synuclein (brown bars) for patients 1-8 in both the DLBTau^-^ and DLBTau^+^ subgroups. Six out of the eight DLBTau^+^ patients showed higher percentage of tau positive tissue than DLBTau^-^ patients. DLBTau^-^ patients 7 and 8 had higher percentages of pTau positive tissue than 2 DLBTau^+^ patients (1 and 2). A-syn percentage varied between the two subgroups but was consistently higher than pTau in all patients except in DLBTau^+^ patient 8.
