# Supplementary material for: Dementia with lewy bodies patients with high tau levels display unique proteome profiles
Source: Mol Neurodegener. 2024 Dec 19;19:98. doi: 10.1186/s13024-024-00782-0 (PMC11657859; doi:10.1186/s13024-024-00782-0)
Supplement: Supplementary file 1 — Supplementary Material 1. [file 13024_2024_782_MOESM1_ESM.zip › Supplementary Figure 3.docx]

Supplementary Figure 3


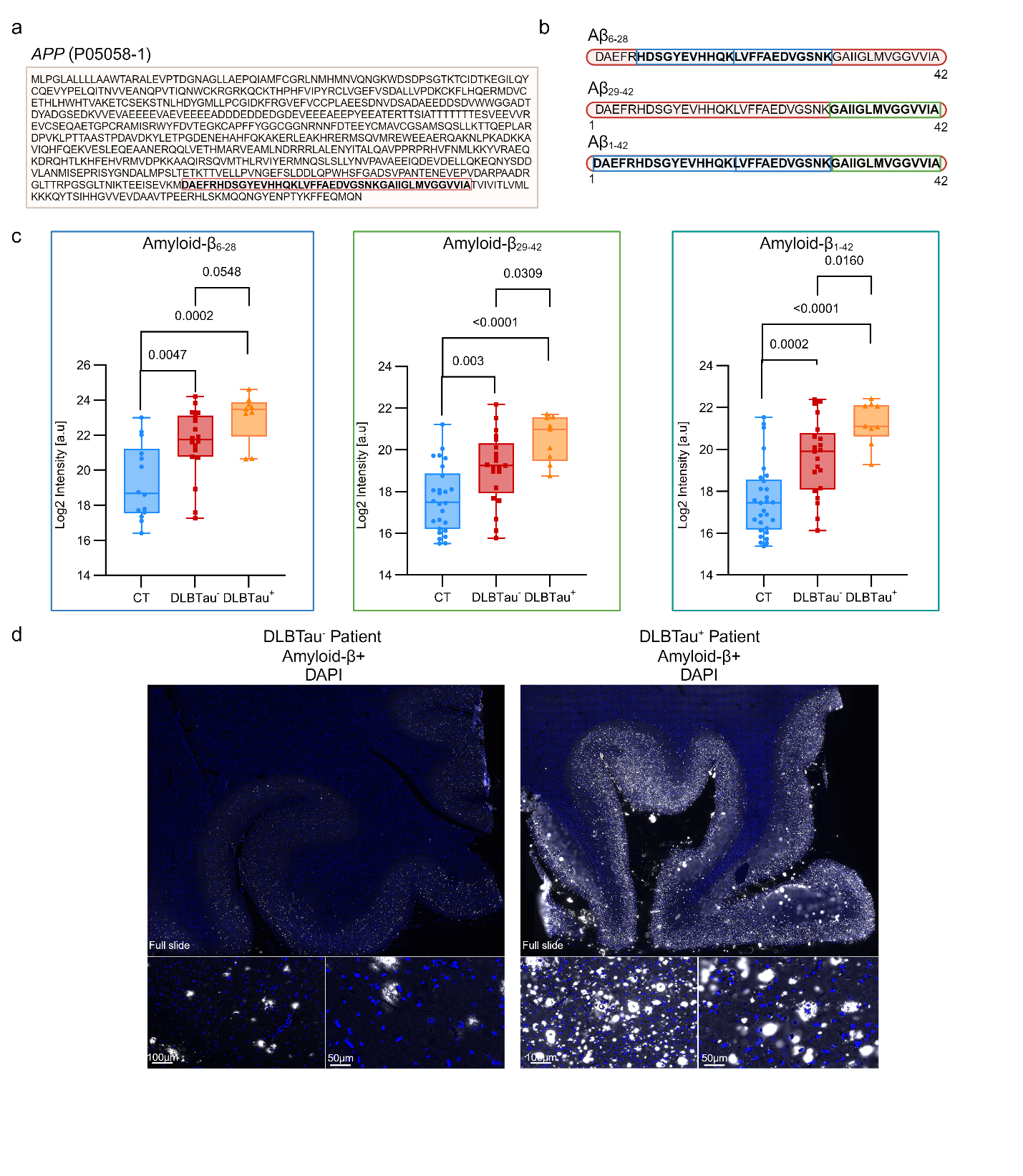


**Supplementary Figure 3. Intensities of amyloid-β peptides are higher in DLBTau^+^ compared to controls and DLBTau^-^.** (a) The sequence of the 770 amino acid APP protein is displayed, highlighting the amyloid-β region. (b) The amyloid-β region shows the identified tryptic (blue) and semi-tryptic (green) peptides. Three peptide sets were used for quantification: Aβ_6-28_, Aβ_29-42,_ and Aβ_1-42_. (c) Box-and-whisker plots display the Log2-transformed intensities of Aβ_6-28_ (comprising two tryptic peptides), Aβ_29-42_ (comprising a semi-tryptic peptide ending at position 42), and Aβ_1-42_ (comprising both tryptic and semi-tryptic peptides). The analysis reveals significant upregulation of amyloid-β in both DLBTau⁻ and DLBTau⁺ groups compared to controls. Two peptide sets (Aβ_29-42_ and Aβ_1-42_) show significant upregulation in DLBTau⁺ compared to DLBTau⁻. (d) Multiplex immunofluorescence of cortical tissue highlights DAPI-stained nuclei (blue) and amyloid-β (white). Lower amyloid-β staining is observed in the DLBTau⁻ patient compared to the DLBTau⁺ patient. Images are presented at x20 magnification with full-slide, 100 μm, and 50 μm views.
