# Supplementary material for: Dementia with lewy bodies patients with high tau levels display unique proteome profiles
Source: Mol Neurodegener. 2024 Dec 19;19:98. doi: 10.1186/s13024-024-00782-0 (PMC11657859; doi:10.1186/s13024-024-00782-0)
Supplement: Supplementary file 1 — Supplementary Material 1. [file 13024_2024_782_MOESM1_ESM.zip › Supplementary Figure 4.docx]

Supplementary Figure 4


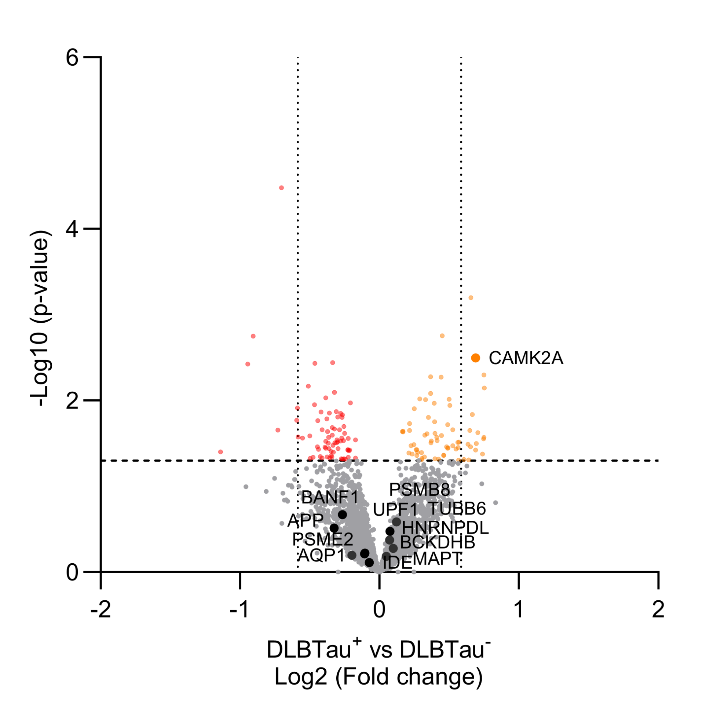


**Supplementary Figure 4. Proteomic changes of the soluble fraction in DLBTau^+^ versus DLBTau^-^ patients.** Volcano plot depicting the up and down-regulated proteins in the soluble fractions from the cortical tissues of the DLBTau^-^ (red) and the DLBTau^+^ (orange) patients versus each other. Each diagram delineates the positioning and significance of the previously identified proteins within the insoluble fraction, reflecting their presence in the soluble fractions. Lines show cut-off -Log10 p-value of 1.3 (p-value <0.05), and Log2 fold change of > 0.585 and < -0.585 for fold change.
