# Supplementary material for: Dementia with lewy bodies patients with high tau levels display unique proteome profiles
Source: Mol Neurodegener. 2024 Dec 19;19:98. doi: 10.1186/s13024-024-00782-0 (PMC11657859; doi:10.1186/s13024-024-00782-0)
Supplement: Supplementary file 1 — Supplementary Material 1. [file 13024_2024_782_MOESM1_ESM.zip › Supplementary Figure 5 .docx]

Supplementary Figure 5


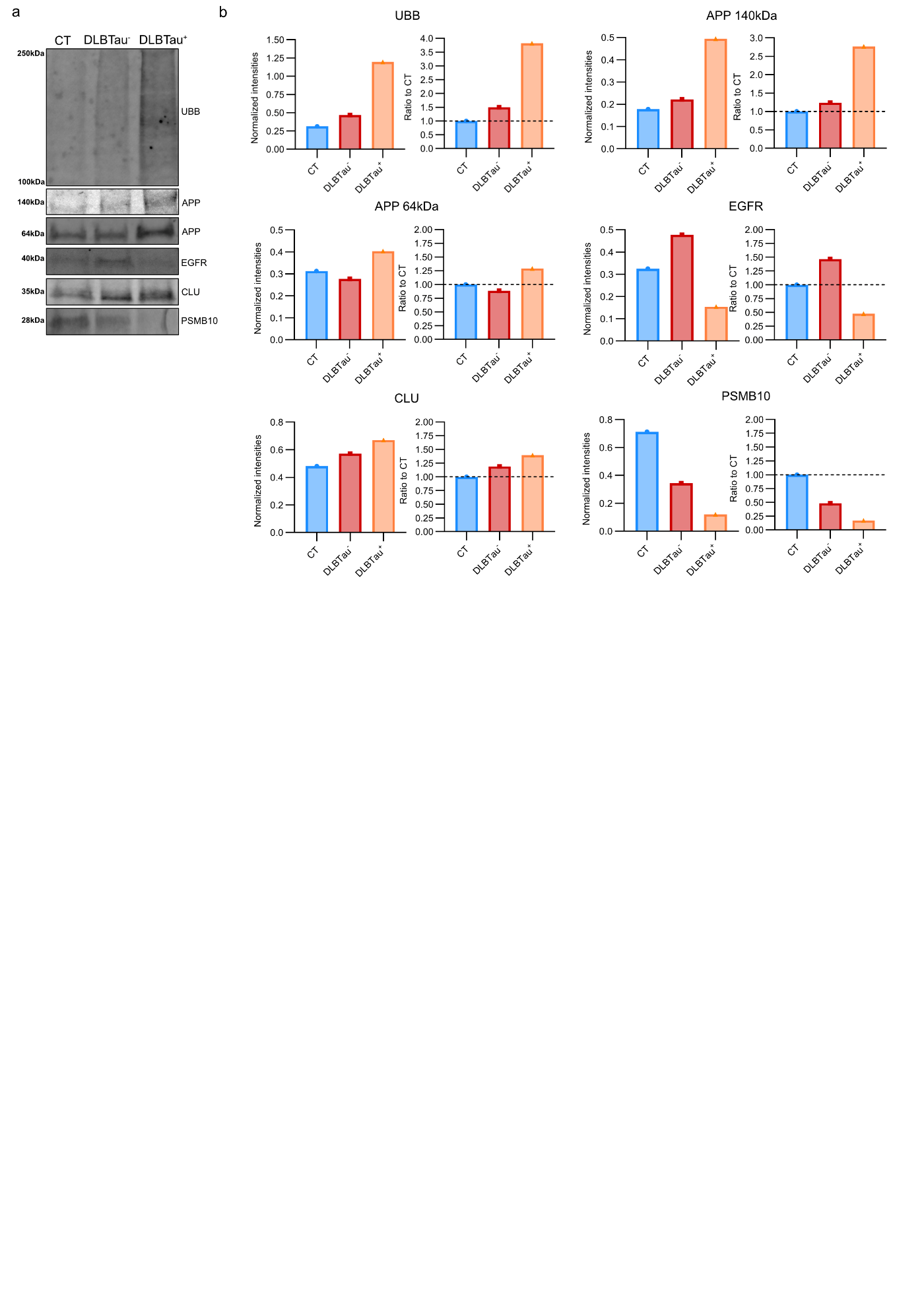


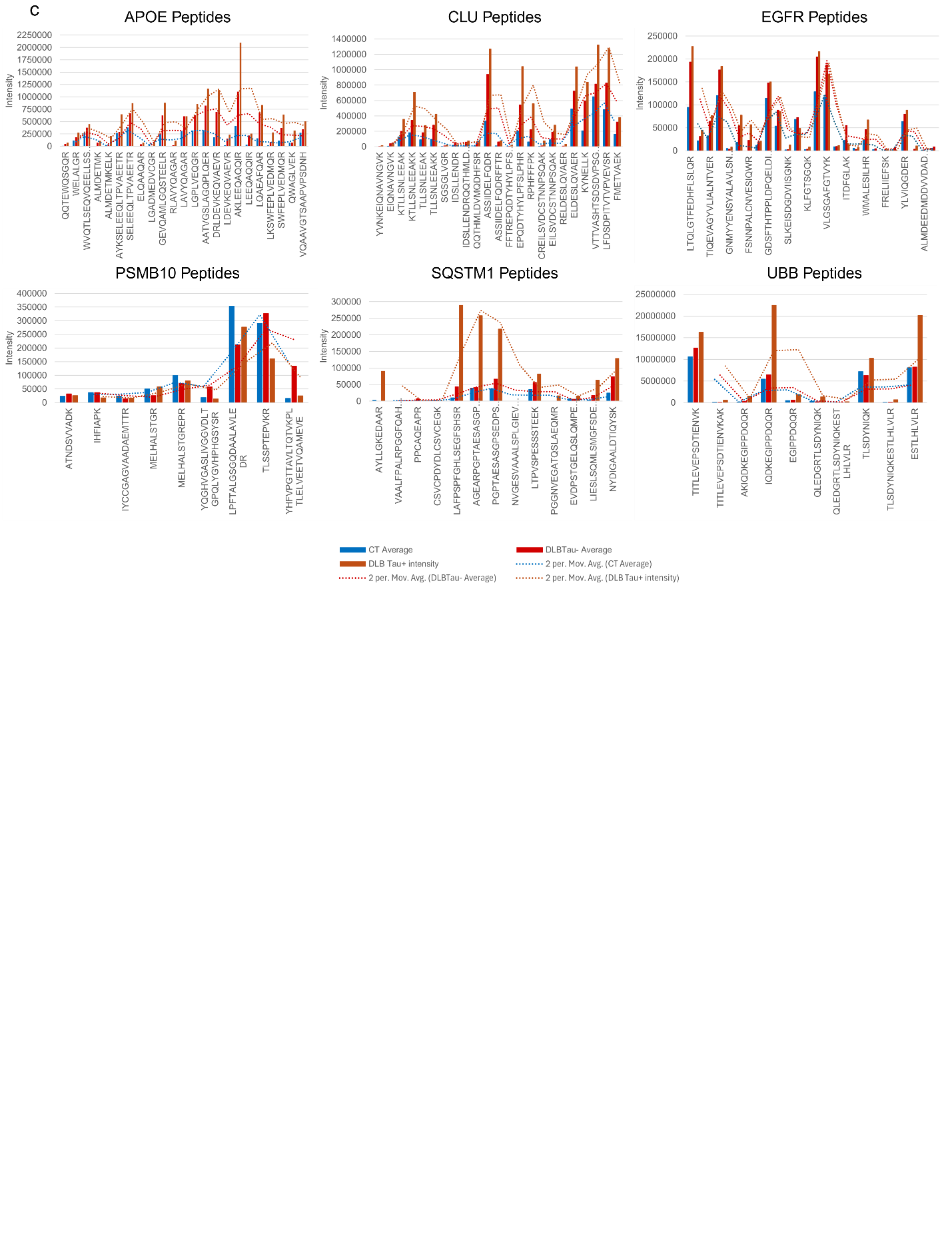


**Supplementary Figure 5. Western blot and peptide analysis shows similar trends for proteins UBB, APP,APOE, EGFR, CLU, SQSTM1 and PSMB10 in Control, DLBTau⁻, and DLBTau⁺ Groups.** (a) Western blot images showing the protein expression levels of UBB, APP (140kDa and 64kDa isoforms), EGFR, CLU, and PSMB10 across pooled samples from CT, DLBTau⁻, and DLBTau⁺ patient groups. Molecular weight markers are shown to the left. (b) Bar plots representing the normalized intensities (left panels) and the ratio of normalized intensities to control (CT) (right panels) for each protein. Each bar represents a single value derived from the pooled sample of patients per group (Control, DLBTau⁻, and DLBTau⁺). The right panels depict the relative fold change in intensity for DLBTau⁻ and DLBTau⁺ compared to the control group (CT), with the control set at a ratio of 1 (dashed line). (c) Average intensity per CT, DLBTau^-^ and DLBTau^+^ patients per peptide identified in proteins of interest: APOE, CLU, EGFR, PSMB10, SQSTM1 and UBB. 2-period moving average trendlines are shown for each patient group.
