# Supplementary material for: Dementia with lewy bodies patients with high tau levels display unique proteome profiles
Source: Mol Neurodegener. 2024 Dec 19;19:98. doi: 10.1186/s13024-024-00782-0 (PMC11657859; doi:10.1186/s13024-024-00782-0)
Supplement: Supplementary file 1 — Supplementary Material 1. [file 13024_2024_782_MOESM1_ESM.zip › Supplementary Figure 6.docx]

Supplementary Figure 6


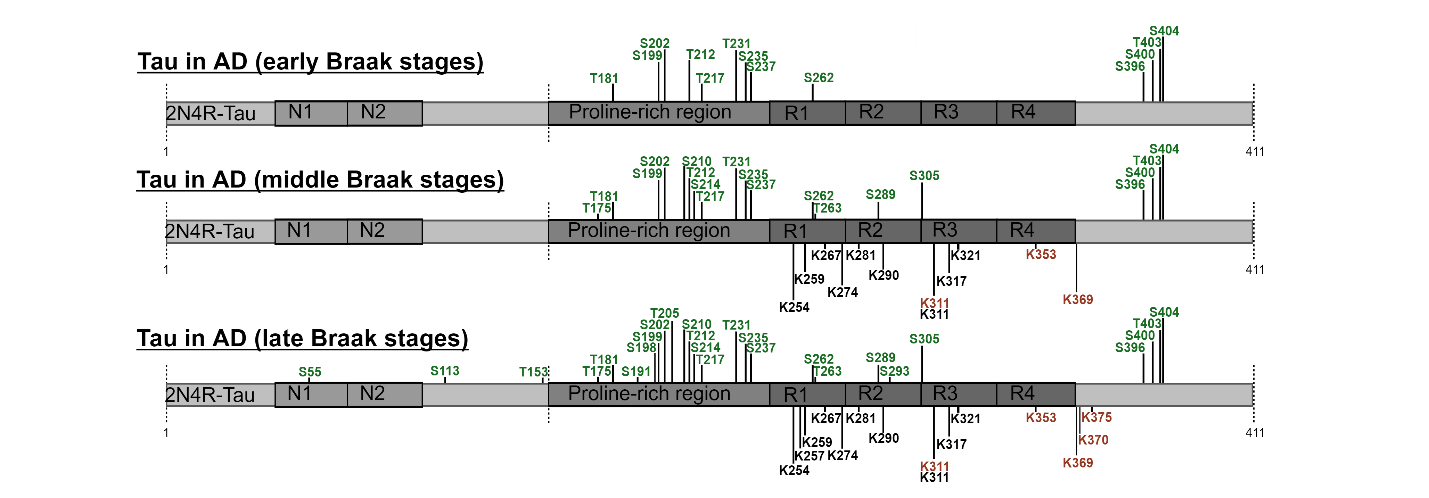


**Supplementary Figure 6. PTM profiles of tau in early, middle, and late Braak stages of AD subjects.** Cumulative map of post-translational modifications (PTMs) identified on tau in AD patients at different Braak stages, as determined in a previous study. Early-stage AD shows phosphorylation primarily in the proline-rich region and C-terminal. Middle-stage AD includes additional phosphorylation and ubiquitination sites. Late-stage AD is characterized by extensive phosphorylation across the tau protein, including the N-terminus, with increased ubiquitination and the appearance of acetylation sites.
