# Supplementary material for: Dementia with lewy bodies patients with high tau levels display unique proteome profiles
Source: Mol Neurodegener. 2024 Dec 19;19:98. doi: 10.1186/s13024-024-00782-0 (PMC11657859; doi:10.1186/s13024-024-00782-0)
Supplement: Supplementary file 2 — Supplementary Material 2. [file 13024_2024_782_MOESM2_ESM.zip › Supplementary Table S1.docx]

Supplementary Table S1

| **Patient Cohort** | **Total number of patients** | **Age (years)**  **Mean** | **Age (years)**  **Range** | **PMI (hours)**  **Mean** | **PMI (hours)**  **Range** | **Sex**  **Male: Female** |
| --- | --- | --- | --- | --- | --- | --- |
| Controls | 29 | 71 | 50-100 | 22.3 | 6.17-30.6 | 19:11 |
| DLBTau^-^ | 21 | 74 | 58-86 | 19.6 | 5.88-31.25 | 15: 6 |
| DLBTau^+^ | 9 | 80 | 65-89 | 21.8 | 11.85-29.75 | 7: 2 |

**Supplementary Table S1: Summary of demographic and postmortem characteristics of the patient cohort.** The table includes the total number of patients, mean age, age range, mean postmortem interval (PMI), PMI range, and the male-to-female ratio for controls, DLBTau^-^, and DLBTau^+^ subgroups.
