# Supplementary material for: Dementia with lewy bodies patients with high tau levels display unique proteome profiles
Source: Mol Neurodegener. 2024 Dec 19;19:98. doi: 10.1186/s13024-024-00782-0 (PMC11657859; doi:10.1186/s13024-024-00782-0)
Supplement: Supplementary file 2 — Supplementary Material 2. [file 13024_2024_782_MOESM2_ESM.zip › Supplementary Table S2.docx]

Supplementary Table S2

| **Sample ID** | **DLB Subgroup** | **PMI** | **Age** | **Sex** | **Brain Region** | **Lewy Body Stage** | **Braak Stage** | **Amyloid-β Log2 Intensity** | **Neuropathology** |
| --- | --- | --- | --- | --- | --- | --- | --- | --- | --- |
| CT1.1 | N/A | 23.5 | 71 | Male | Dorsolateral Prefrontal cortex (BA9) | N/A | I | 15.7 | 1. Neurofibrillary degeneration, Braak and Braak stage I. 2. Small vessel cerebrovascular disease with atherosclerosis, arteriosclerosis, arteriolosclerosis, and a lacune in the hippocampus. 3. Autolysis, moderate, with mild acute ischemia. |
| CT1.2 | N/A | 19.22 | 63 | Male | Dorsolateral Prefrontal cortex (BA9) | N/A | I | 21.2 | 1. Neurofibrillary degeneration, Braak and Braak stage I, with non-neuritic neocortical amyloid plaques. 2. Arteriosclerosis, neostriatum. 3. Vasculitic (lipohyalinosis), arteriole in globus pallidus. |
| CT1.3 | N/A | 17.65 | 68 | Male | Dorsolateral Prefrontal cortex (BA9) | N/A | I | 21 | 1. Central pontine hemorrhage, acute. 2. Atherosclerosis, severe and arteriosclerosis. 3. Neurofibrillary degeneration, Braak stage I, with non-neuritic neocortical amyloid plaques and amyloid angiopathy. |
| CT2.1 | N/A | 24.17 | 53 | Male | Dorsolateral Prefrontal cortex (BA9) | N/A | I | 17.4 | 1. Neurofibrillary degeneration, Braak and Braak stage I. 2. Mild arteriosclerosis, basal ganglia. |
| CT2.2 | N/A | 22.6 | 59 | Male | Dorsolateral Prefrontal cortex (BA9) | N/A | - | 17.1 | 1. Mild arteriosclerosis. 2. Acute microinfarct, head of caudate nucleus. 3. Ischemia, neurons of Sommer's sector, hippocampus. 4. Sparse non-neuritic amyloid plaques, cerebral neocortex. |
| CT2.3 | N/A | 21.13 | 69 | Male | Dorsolateral Prefrontal cortex (BA9) | N/A | - | 20.1 | 1. No Neuropathological abnormality |
| CT3.1 | N/A | 24.13 | 50 | Male | Dorsolateral Prefrontal cortex (BA9) | N/A | I | 16.1 | 1. Neurofibrillary tangle, basal nucleus of Meynert, Braak stage I. |
| CT3.2 | N/A | 26.3 | 58 | Male | Dorsolateral Prefrontal cortex (BA9) | N/A | I | 15.8 | 1. Neurofibrillary degeneration, Braak and Braak stage I, with non-neuritic neocortical amyloid plaques. 2. Atherosclerosis and arteriosclerosis. |
| CT3.3 | N/A | 22 | 71 | Female | Dorsolateral Prefrontal cortex (BA9) | N/A | I | 16 | 1. Neurofibrillary tangles, Braak stage I. 2. Atherosclerosis, severe, and mild arteriosclerosis. |
| CT4.1 | N/A | 23.58 | 63 | Female | Dorsolateral Prefrontal cortex (BA9) | N/A | I | 18.5 | 1. Neurofibrillary degeneration, Braak and Braak stage I, with rare to sparse non-neuritic neocortical amyloid plaques. 2. Atherosclerosis and arteriosclerosis. |
| CT4.2 | N/A | 22.93 | 79 | Male | Dorsolateral Prefrontal cortex (BA9) | N/A | I | 18.1 | 1. Neurofibrillary degeneration, Braak and Braak stage I. 2. Atherosclerosis and arteriosclerosis, and a possible microinfarct in the nucleus accumbens |
| CT4.3 | N/A | 11.02 | 55 | Male | Dorsolateral Prefrontal cortex (BA9) | N/A | - | 17.6 | 1. No neuropathological abnormality. 2. Acute hypoxic/ischemic encephalopathy. 3. cerebral arteriolosclerosis, mild to moderate. |
| CT5.1 | N/A | 28.12 | 56 | Male | Dorsolateral Prefrontal cortex (BA9) | N/A | - | 17.5 | 1. No neuropathological abnormality. 2. Cerebral arteriolosclerosis, mild to moderate. 3. Acute anoxic/hypoxic encephalopathy. |
| CT5.2 | N/A | 18.30 | 80 | Male | Dorsolateral Prefrontal cortex (BA9) | N/A | I | 18.1 | 1. Demyelinated plaque, white matter adjacent to lateral ventricle. 2. Neurofibrillary degeneration, Braak stage I. 3. Atherosclerosis and arteriosclerosis. |
| CT5.3 | N/A | 25.23 | 77 | Male | Dorsolateral Prefrontal cortex (BA9) | N/A | II | 21.5 | 1. Neurofibrillary degeneration, Braak stage II, with non-neuritic amyloid plaques |
| CT6.1 | N/A | 20.85 | 66 | Male | Dorsolateral Prefrontal cortex (BA9) | N/A | I | 16.9 | 1. Argyrophilic grain disease, mild. 2. Neurofibrillary degeneration, Braak and Braak stage I, with rare non-neuritic neocortical amyloid plaques. 3. Arteriosclerosis, mild. |
| CT6.2 | N/A | 19.30 | 73 | Male | Dorsolateral Prefrontal cortex (BA9) | N/A | I | 15.4 | 1. Purkinje cell loss, cerebellar cortex, with neuron loss in inferior olive (history of epilepsy). 2. Neurofibrillary degeneration, Braak stage I. 3. Atherosclerosis and arteriosclerosis. |
| CT6.3 | N/A | 28.07 | 58 | Male | Dorsolateral Prefrontal cortex (BA9) | N/A | I | 17.5 | 1. Neurofibrillary degeneration, early Braak and Braak stage I, with non-neuritic neocortical amyloid plaques and rare amyloid angiopathy. 2. Severe autolytic changes |
| CT6.4 | N/A | 6.17 | 100 | Male | Dorsolateral Prefrontal cortex (BA9) | N/A | II | 16.5 | 1. Neurofibrillary degeneration, Braak and Braak stage II, with mild amyloid angiopathy. 2. Mild neuron loss, without neurofibrillary tangles, locus coeruleus. 3. Small vessel cerebrovascular disease with atherosclerosis, arteriolosclerosis. |
| CT7.2 | N/A | 20.3 | 71 | Female | Dorsolateral Prefrontal cortex (BA9) | N/A | I | 16.2 | 1. Cerebrovascular disease, with severe atherosclerosis, mild to moderate arteriosclerosis, and recent hemorrhagic infarcts in cerebral white matter and cerebellar cortex. 2. Neurofibrillary tangles, Braak stage I |
| CT7.3 | N/A | 29.18 | 86 | Female | Dorsolateral Prefrontal cortex (BA9) | N/A | - | 17.7 | 1. Mild small vessel cerebrovascular disease with atherosclerosis, arteriosclerosis, arteriolosclerosis, a microinfarct in the putamen, and mineralization of blood vessel walls in the globus pallidus, dentate gyrus, and cerebellar white matter. |
| CT7.4 | N/A | 24.35 | 63 | Male | Dorsolateral Prefrontal cortex (BA9) | N/A | I | 15.5 | 1. Neurofibrillary degeneration, Braak stage I. 2. Atherosclerosis and arteriosclerosis. |
| CT7.5 | N/A | 29.63 | 92 | Female | Dorsolateral Prefrontal cortex (BA9) | N/A | - | 16.6 | 1. Small vessel cerebrovascular disease with atherosclerosis, arteriosclerosis, arteriolosclerosis, cribriform state in superior neostriatum, globus pallidus and thalamus, small remote infarcts in temporal cortex and rostral caudate nucleus and microinfarcts. |
| CT7.6 | N/A | 28.15 | 60 | Female | Dorsolateral Prefrontal cortex (BA9) | N/A | I | 18.7 | 1. Neurofibrillary degeneration, Braak and Braak early stage I, with moderate non-neuritic neocortical amyloid plaques. 2. Atherosclerosis and arteriosclerosis. |
| CT8.1 | N/A | 14.55 | 77 | Male | Dorsolateral Prefrontal cortex (BA9) | N/A | II | 19.1 | 1. Neurofibrillary degeneration, Braak and Braak stage II, with non-neuritic neocortical amyloid plaques. 2. Atherosclerosis and arteriosclerosis. |
| CT8.2 | N/A | 25.85 | 95 | Female | Dorsolateral Prefrontal cortex (BA9) | N/A | II | 18.6 | Cerebrovascular disease: - remote microinfarcts - moderate atherosclerosis - moderate arteriosclerosis Hippocampal sclerosis. Neurofibrillary tangles, Braak and Braak stage II. |
| CT8.3 | N/A | 30.6 | 85 | Female | Dorsolateral Prefrontal cortex (BA9) | N/A | - | 15.5 | No diagnostic abnormalities Comment: This was an 85-year-old woman with a history of lung cancer. There was no evidence of metastatic disease to the brain |
| CT8.4 | N/A | 18 | 93 | Female | Dorsolateral Prefrontal cortex (BA9) | N/A | I | 16.9 | 1. Neurofibrillary degeneration, Braak and Braak stage I. 2. Atherosclerosis and arteriosclerosis, with periarterial neuron loss in neostriatum |
| DLB1.1 | DLBTau^-^ | 29.38 | 84 | Female | Dorsolateral Prefrontal cortex (BA9) | Diffuse LB Disease (Cortical Stage) | II | 20.9 | 1. Diffuse Lewy body disease (dementia with Lewy bodies), cortical stage. 2. Neurofibrillary degeneration, Braak stage II, with non-neuritic neocortical amyloid plaques. 3. Arteriosclerosis. |
| DLB1.2 | DLBTau^-^ | 11.25 | 86 | Male | Dorsolateral Prefrontal cortex (BA9) | Cortical Stage | II | 20.2 | 1. Dementia with Lewy bodies, cortical stage (equivalent to Parkinson disease Braak and Del Tredici stage 5). 2. Neurofibrillary degeneration, Braak and Braak stage II, with neocortical amyloid plaques. 3. Atherosclerosis and arteriosclerosis. |
| DLB1.3 | DLBTau^-^ | 26.61 | 69 | Male | Dorsolateral Prefrontal cortex (BA9) | Late Limbic Stage | - | 18 | 1. Dementia with Lewy bodies, late limbic stage. 2. Sparse non-neuritic neocortical amyloid plaques. 3. Mild arteriosclerosis. |
| DLB2.1 | DLBTau^-^ | 8 | 67 | Male | Dorsolateral Prefrontal cortex (BA9) | Limbic Stage | V | 22.3 | 1. Alzheimer disease, Braak and Braak stage V. 2. Dementia with Lewy bodies, limbic stage. 3. Atherosclerosis and arteriosclerosis, mild. |
| DLB2.2 | DLBTau^-^ | 21.05 | 70 | Male | Dorsolateral Prefrontal cortex (BA9) | Cortical Stage | III-IV | 18.1 | 1. Dementia with Lewy bodies, cortical stage. 2. Early Alzheimer disease, Braak and Braak stage III-IV. 3. Atherosclerosis and arteriosclerosis. Comment: Dementia with Lewy bodies is the pathology responsible for cognitive impairment. The cortical stage. |
| DLB2.3 | DLBTau^+^ | 29.75 | 81 | Male | Dorsolateral Prefrontal cortex (BA9) | Limbic Stage | II | 21.1 | 1. Dementia with Lewy bodies, limbic stage. 2. Neurofibrillary degeneration, Braak and Braak stage II, with non-neuritic neocortical amyloid plaques and amyloid angiopathy. 3. Argyrophilic grain disease, mild. 4. Autolysis, moderate. |
| DLB3.1 | DLBTau^-^ | 5.88 | 79 | Male | Dorsolateral Prefrontal cortex (BA9) | Early Cortical Stage | II | 20 | 1. Diffuse Lewy body disease, early cortical stage. 2. Severe myelinated fiber loss, anterior commissure (temporal limb). 3. Neurofibrillary degeneration, Braak stage II, with non-neuritic neocortical amyloid plaques. 4. Arteriosclerosis. |
| DLB3.2 | DLBTau^-^ | 31.25 | 58 | Female | Dorsolateral Prefrontal cortex (BA9) | Cortical Stage | II | 20.1 | 1. Dementia with Lewy bodies, cortical stage. 2. Neurofibrillary degeneration, Braak and Braak stage II, with amyloid angiopathy and non-neuritic neocortical amyloid plaques. 3. Optic tract myelinated fiber loss, partial. |
| DLB3.3 | DLBTau^-^ | 17.33 | 75 | Male | Dorsolateral Prefrontal cortex (BA9) | Limbic Stage | II | 19 | 1. Diffuse Lewy body disease, limbic stage. 2. Neurofibrillary degeneration, Braak stage II. 3. Severe amyloid angiopathy, with perivascular amyloid deposits. 4. Arteriosclerosis, with microinfarcts and an organized vascular thrombus. |
| DLB4.1 | DLBTau^-^ | 25.23 | 85 | Male | Dorsolateral Prefrontal cortex (BA9) | Early Limbic Stage | I | 17.4 | 1. Synuclein-positive pathology consistent with early limbic stage dementia with Lewy bodies, or early stage 4 Parkinson’s disease. 2. Neurofibrillary degeneration, Braak and Braak stage I, with amyloid angiopathy. |
| DLB4.2 | DLBTau^-^ | 25 | 72 | Female | Dorsolateral Prefrontal cortex (BA9) | Early Cortical Stage | II-III | 20.6 | 1. Dementia with Lewy bodies early cortical stage, or Parkinson disease, Braak and Del Tredici early stage 5. 2. Neurofibrillary degeneration, Braak and Braak stage II to III, with severe amyloid angiopathy and abundant non-neuritic neocortical amyloid plaques. |
| DLB4.3 | DLBTau^+^ | 22 | 85 | Male | Dorsolateral Prefrontal cortex (BA9) | Cortical Stage | IV | 22.4 | 1. Diffuse Lewy body disease, cortical stage. 2. Early Alzheimer disease, Braak stage IV, with severe amyloid angiopathy. 3. Cerebral contusion, frontal pole cortex (plaque jaune). 4. Capillary telangiectasias, basis pontis. 5. Atherosclerosis, arteriosclerosis. |
| DLB5.1 | DLBTau^-^ | 20.75 | 71 | Male | Dorsolateral Prefrontal cortex (BA9) | Cortical Stage | II | 19.6 | 1. Dementia with Lewy bodies, cortical stage. 2. Neurofibrillary degeneration, Braak and Braak stage II, with amyloid plaques. 3. Arteriosclerosis and mild atherosclerosis. 4. Tentorial notching of ventral uncus, consistent with terminal brain swelling. |
| DLB5.2 | DLBTau^+^ | 20.67 | 81 | Female | Dorsolateral Prefrontal cortex (BA9) | Cortical Stage | III | 21 | 1. Diffuse Lewy body disease (dementia with Lewy bodies), cortical stage. 2. Early Alzheimer disease, Braak stage III. 3. Atherosclerosis and arteriosclerosis. 4. Mild ischemic changes, pyramidal cells of Sommer's sector, hippocampus. 5. Mild loss of cerebellum. |
| DLB5.3 | DLBTau^+^ | 29.53 | 72 | Male | Dorsolateral Prefrontal cortex (BA9) | Cortical Stage | V | 20.3 | 1. Dementia with Lewy bodies, cortical stage. 2. Alzheimer disease, early Braak and Braak stage V, with mild amyloid angiopathy. 3. Atherosclerosis and arteriosclerosis. |
| DLB6.1 | DLBTau^-^ | 14.41 | 69 | Male | Dorsolateral Prefrontal cortex (BA9) | Limbic Stage | III | 18.9 | 1. Diffuse Lewy body disease, limbic stage. 2. neurofibrillary degeneration, Braak stage III, with non-neuritic neocortical amyloid plaques and amyloid angiopathy. |
| DLB6.2 | DLBTau^+^ | 21.35 | 65 | Male | Dorsolateral Prefrontal cortex (BA9) | Cortical Stage | III-IV | 21 | 1. Dementia with Lewy bodies, cortical stage. 2. Early Alzheimer disease, Braak and Braak stage III to IV, with hippocampal sparing. 3. Atherosclerosis and arteriosclerosis. 4. Autolysis. |
| DLB6.3 | DLBTau^-^ | 12.75 | 62 | Male | Dorsolateral Prefrontal cortex (BA9) | Limbic Stage | II | 19.2 | 1. Diffuse Lewy body disease, limbic stage. 2. Argyrophilic grain disease, mild. 3. Neurofibrillary degeneration, Braak stage II, with non-neuritic neocortical amyloid plaques and amyloid angiopathy. 4. Atherosclerosis and arteriosclerosis. |
| DLB7.1 | DLBTau^-^ | 22.61 | 71 | Male | Dorsolateral Prefrontal cortex (BA9) | Limbic Stage | II | 16.1 | 1. Dementia with Lewy bodies, limbic stage. 2. Neurofibrillary degeneration, Braak and Braak stage II. 3. Small vessel cerebrovascular disease with atherosclerosis, arteriosclerosis, arteriolosclerosis and microinfarcts in temporal cortex, hippocampus. |
| DLB7.2 | DLBTau^+^ | 11.85 | 85 | Male | Dorsolateral Prefrontal cortex (BA9) | Cortical Stage | V | 22.1 | 1. Dementia with Lewy bodies, cortical stage. 2. Alzheimer disease, Braak and Braak stage V, with severe amyloid angiopathy. 3. Atherosclerosis and arteriosclerosis. |
| DLB7.3 | DLBTau^+^ | 20.33 | 82 | Female | Dorsolateral Prefrontal cortex (BA9) | Cortical Stage | V | 22 | 1. Dementia with Lewy bodies, cortical stage. 2. Alzheimer disease, Braak and Braak stage V, with amyloid angiopathy. 3. Mild argyrophilic grain disease. 4. Atherosclerosis and arteriosclerosis. |
| DLB8.1 | DLBTau^-^ | 21.25 | 79 | Male | Dorsolateral Prefrontal cortex (BA9) | Cortical Stage | II | 22.2 | 1. Dementia with Lewy bodies, cortical stage. 2. Neurofibrillary degeneration, Braak and Braak stage II, with non-neuritic neocortical amyloid plaques. Comment: Dementia with Lewy bodies is the pathology responsible for cognitive impairment. |
| DLB8.2 | DLBTau^+^ | 16.83 | 80 | Male | Dorsolateral Prefrontal cortex (BA9) | Cortical Stage | V | 19.3 | 1. Dementia with Lewy bodies, cortical stage. 2. Alzheimer disease, early Braak and Braak stage V. 3. Atherosclerosis and arteriosclerosis. |
| DLB8.3 | DLBTau^+^ | 23.75 | 89 | Male | Dorsolateral Prefrontal cortex (BA9) | Cortical Stage | V | 22.1 | 1. Dementia with Lewy bodies, cortical stage ( Braak and Del Tredici stage 5). 2. Alzheimer disease, hippocampal sparing type. 3. Minute arterial angioma, cerebellar white matter. 4. Tentorial notching of ventral uncus, consistent with terminal brain swelling. |
| DLB9.1 | DLBTau^-^ | 16.5 | 77 | Male | Dorsolateral Prefrontal cortex (BA9) | Cortical Stage | II | 20.5 | 1. Dementia with Lewy bodies, cortical stage. 2. Neurofibrillary degeneration, Braak and Braak stage II, with non-neuritic and rare neuritic neocortical amyloid plaques. 3. Arteriosclerosis, striatum. |
| DLB9.2 | DLBTau^-^ | 12.25 | 85 | Female | Dorsolateral Prefrontal cortex (BA9) | Limbic Stage | V | 22.4 | 1. Diffuse Lewy body disease, limbic stage. 2. Alzheimer disease, Braak stage V, with amyloid angiopathy. 3. Small vessel cerebrovascular disease with atherosclerosis, arteriosclerosis, arteriolosclerosis, and a microinfarct in the putamen. |
| DLB9.3 | DLBTau^-^ | 18.28 | 62 | Male | Dorsolateral Prefrontal cortex (BA9) | Cortical Stage | I | 19.9 | 1. Dementia with Lewy bodies, cortical stage. 2. Neurofibrillary degeneration, Braak and Braak stage I, with moderate non-neuritic and rare neuritic amyloid plaques. 3. Mild atherosclerosis. |
| DLB9.4 | DLBTau^-^ | 23.66 | 79 | Male | Dorsolateral Prefrontal cortex (BA9) | Cortical Stage | II | 21.8 | 1. Dementia with Lewy bodies, cortical stage. 2. Neurofibrillary degeneration, Braak and Braak stage II, with non-neuritic neocortical amyloid plaques and amyloid angiopathy. |
| DLB9.5 | DLBTau^-^ | 25.67 | 72 | Female | Dorsolateral Prefrontal cortex (BA9) | Limbic Stage | II | 17.7 | 1. Dementia with Lewy bodies, limbic stage. 2. Neurofibrillary degeneration, Braak and Braak stage II. 3. Mild arteriosclerosis (globus pallidus). 4. Autolysis, moderate. 5. Tentorial notching of ventral uncus, consistent with terminal brain swelling. |
| DLB9.6 | DLBTau^-^ | 23.22 | 76 | Female | Dorsolateral Prefrontal cortex (BA9) | Diffuse Cortical Lewy Body Disease | III-IV/VI | 16.7 | 1. Diffuse Cortical Lewy Body Disease. 2. Neurofibrillary degeneration, Braak stage III-IV/VI. 3. Cerebral arteriolosclerosis, moderate. 4. Remote thalamic infarct, left. |

**Supplementary Table S2: Patient Demographics and Neuropathological Details.** Information includes age, sex, postmortem interval (PMI), DLB subgrouping (DLBTau^-^ and DLBTau^+^), Lewy Body stage, Braak stage, Amyloid-β Log2 intensity (calculated by mass spectrometry) and additional neuropathological characteristics. All subjects were included in the analyses conducted in the study.
