# Supplementary material for: Dementia with lewy bodies patients with high tau levels display unique proteome profiles
Source: Mol Neurodegener. 2024 Dec 19;19:98. doi: 10.1186/s13024-024-00782-0 (PMC11657859; doi:10.1186/s13024-024-00782-0)
Supplement: Supplementary file 2 — Supplementary Material 2. [file 13024_2024_782_MOESM2_ESM.zip › Supplementary Table S3.docx]

**Table S3- Modified peptide sequences of α-synuclein and tau with subgroup frequency**

| ***Α-synuclein Modification*** | ***a.a. site*** | ***DLBTau- Patient Freq. (%)*** | ***DLBTau+ Patient Freq. (%)*** |
| --- | --- | --- | --- |
| Acetylation | K12 | 19 | 11 |
| Phosphorylation | T22 | 5 | 0 |
| Ubiquitination | K23 | 0 | 22 |
| Phosphorylation | T33 | 10 | 0 |
| Acetylation | K34 | 19 | 0 |
| Phosphorylation | S42 | 5 | 0 |
| Phosphorylation | T44 | 5 | 11 |
| Acetylation | K45 | 19 | 11 |
| Phosphorylation | T54 | 10 | 0 |
| Acetylation | K58 | 10 | 11 |
| Phosphorylation | T59 | 10 | 0 |
| Acetylation | K60 | 29 | 44 |
| Phosphorylation | T64 | 10 | 0 |
| Phosphorylation | T72 | 5 | 0 |
| Phosphorylation | T75 | 5 | 0 |
| Acetylation | K80 | 0 | 11 |
| Phosphorylation | T81 | 10 | 0 |
| Phosphorylation | T92 | 5 | 0 |
| Phosphorylation | S129 | 5 | 0 |
| ***Tau Modification*** | ***a.a. site*** | ***DLBTau- Patient Freq. (%)*** | ***DLBTau+ Patient Freq. (%)*** |
| Phosphorylation | S46 | 22 | 5 |
| Phosphorylation | T111 | 11 | 0 |
| Phosphorylation | T123 | 11 | 0 |
| Phosphorylation | T175 | 22 | 5 |
| Phosphorylation | T181 | 100 | 14 |
| Phosphorylation | S185 | 11 | 0 |
| Phosphorylation | S191 | 11 | 0 |
| Phosphorylation | S199 | 22 | 0 |
| Phosphorylation | S202 | 100 | 67 |
| Phosphorylation | T212 | 56 | 0 |
| Phosphorylation | T217 | 100 | 0 |
| Phosphorylation | T231 | 100 | 0 |
| Phosphorylation | T235 | 67 | 0 |
| Ubiquitination | K254 | 11 | 0 |
| Ubiquitination | K257 | 78 | 0 |
| Phosphorylation | S258 | 44 | 0 |
| Ubiquitination | K259 | 22 | 0 |
| Acetylation | K259 | 11 | 0 |
| Phosphorylation | S262 | 100 | 0 |
| Phosphorylation | T263 | 89 | 0 |
| Ubiquitination | K267 | 33 | 0 |
| Acetylation | K267 | 22 | 0 |
| Ubiquitination | K274 | 11 | 0 |
| Ubiquitination | K281 | 89 | 0 |
| Acetylation | K281 | 22 | 0 |
| Phosphorylation | S289 | 22 | 0 |
| Ubiquitination | K298 | 44 | 0 |
| Acetylation | K298 | 11 | 0 |
| Phosphorylation | S305 | 33 | 0 |
| Ubiquitination | K311 | 56 | 0 |
| Acetylation | K311 | 22 | 0 |
| Acetylation | K317 | 11 | 0 |
| Ubiquitination | K317 | 56 | 0 |
| Ubiquitination | K321 | 11 | 0 |
| Acetylation | K321 | 22 | 0 |
| Phosphorylation | S324 | 33 | 0 |
| Acetylation | K331 | 11 | 0 |
| Ubiquitination | K343 | 11 | 0 |
| Acetylation | K343 | 67 | 0 |
| Acetylation | K347 | 33 | 0 |
| Acetylation | K353 | 78 | 0 |
| Phosphorylation | S356 | 11 | 0 |
| Acetylation | K369 | 22 | 0 |
| Acetylation | K370 | 33 | 0 |
| Ubiquitination | K375 | 22 | 0 |
| Ubiquitination | K385 | 56 | 0 |
| Phosphorylation | T386 | 11 | 0 |
| Phosphorylation | S396 | 78 | 0 |
| Phosphorylation | S400 | 22 | 0 |
| Phosphorylation | T403 | 44 | 24 |
| Phosphorylation | S404 | 89 | 52 |
| Phosphorylation | T414 | 11 | 0 |
| Phosphorylation | S416 | 11 | 0 |
| Phosphorylation | S422 | 22 | 0 |
